# An Intracellular Sensing and Signal Transduction System That Regulates the Metabolism of Polycyclic Aromatic Hydrocarbons in Bacteria

**DOI:** 10.1128/mSystems.00636-21

**Published:** 2021-10-05

**Authors:** Wanpeng Wang, Zongze Shao

**Affiliations:** a Key Laboratory of Marine Genetic Resources, Third Institute of Oceanography, Ministry of Natural Resources, Xiamen, China; b Key Laboratory of Marine Genetic Resources of Fujian Province, Xiamen, China; c Southern Marine Science and Engineering Guangdong Laboratory, Zhuhai, China; Ocean University of China

**Keywords:** *Cycloclasticus*, PAH degradation, intracellular sensing, polycyclic aromatic hydrocarbons, signal transduction system

## Abstract

Many bacteria utilize polycyclic aromatic hydrocarbon (PAH) as carbon and energy sources for growth. These bacteria play an important role in the amelioration of PAH pollution in various environments. However, it is unclear how bacteria sense PAHs and how PAH degradation pathways are regulated via signal transduction. Here, we investigated these mechanisms in *Cycloclasticus*, a ubiquitous PAH-degrading bacterium in marine environments. We identified the key genes involved in intracellular PAH sensing, signal transduction, and the differential regulation of degradation pathways for each PAH examined. Our results showed that PAHs bind specifically to a diguanylate cyclase PdgC, leading to the generation of cyclic dimeric GMP (c-di-GMP), which subsequently binds to two CRP/FNR family regulators, DPR-1 and DPR-2. c-di-GMP activates the transcription of DPR-1 and DPR-2 to positively regulate degradation pathways specific to pyrene and phenanthrene/naphthalene, respectively. This is the first report of an intracellular signal transduction pathway associated with PAH degradation in bacteria. Our results improve our understanding of the intracellular responses to PAHs. The existence of the identified genes in other bacteria indicates that the strategy described here is widely used by other PAH-degrading bacteria.

**IMPORTANCE** Polycyclic aromatic hydrocarbons (PAHs) are widely distributed and have been found indoors, in the atmosphere, in terrestrial soils, in marine waters and sediments, and even in outer space. Bacteria degrade PAHs via degradation pathways. PAH signal sensing and transduction, as well as the regulation of PAH degradation pathways, are crucial for bacterial PAH biodegradation. However, prior to this study, these processes were poorly known. This study employed multiple molecular approaches to better understand the regulatory networks controlling PAH metabolism in bacteria. This report illustrates, for the first time, PAH-specific intracellular sensing, signal transduction, and metabolic regulatory pathways. Our results will help to increase our understanding of the hydrocarbon-metabolism regulatory network as well as the regulatory intricacies that control microbial biodegradation of organic matter. These key data should be considered to improve the rational design and efficiency of recombinant biodegradable, bacterial biosensors, and biocatalysts in modern green chemistry.

## INTRODUCTION

Polycyclic aromatic hydrocarbons (PAHs) are widely distributed, and they have been found indoors, in the atmosphere, in terrestrial soils, in marine waters and sediments, and even in outer space (1–3). Although PAHs are toxic, they can serve as energy and carbon sources for many bacteria and fungi (4) and even some algae (5) and archaea (6). Bacteria may degrade PAHs via degradation pathways, which are usually initiated by a ring-hydroxylating dioxygenase (RHD) under aerobic conditions. They form a *cis*-dihydrodiol, which is rearomatized to a diol intermediate by dehydrogenase (7, 8). These diol intermediates may then be cleaved by intradiol or extradiol ring-cleaving dioxygenases via either ortho-cleavage or meta-cleavage pathways and further oxygenase reactions, producing intermediates, such as catechols, which are ultimately converted into tricarboxylic acid (TCA) cycle intermediates (7, 8).

Environmental sensing and homeostatic regulation are essential functions of living organisms (9). Signaling systems enable cells to recognize ambient and intracellular alterations and to trigger adaptive responses (9, 10). However, the regulatory systems associated with PAH sensing and signal transduction, which regulate PAH degradation pathways, remain poorly understood. To date, a few transcriptional regulators have been found adjacent to PAH catabolic genes, including LysR in Mycobacterium vanbaalenii PYR-1 (11), AraC and GntR in *N. pentaromativorans* US6-1 (12), TetR in *Delftia* sp. strain Cs1-4 (13), MarR in *Alteromonas* sp. strain SN2 (14), and PahR in *Novosphingobium* sp. strain HR1a (15); thus, these regulators may play roles in the regulation of PAH biodegradation. Recently, we identified LysR-like regulators associated with the PAH degradation pathways of the deep-sea, fluoranthene-degrading bacterium Celeribacter indicus P73 (16). However, these regulators were identified, and their function requires further systematic verification.

*Cycloclasticus* bacteria are ubiquitous in marine environments. They even inhabit the remote, unpolluted oceanic areas and dominate as a marine obligate PAH degrader by utilizing various aromatic hydrocarbons (17–20). For example, *Cycloclasticus* sp. strain P1, which was isolated from the deep-sea sediments of the Pacific Ocean, degrades pyrene, a four-ring PAH, in addition to biphenyl, naphthalene, 2-methylnaphthalene, 2,6-dimethylnaphthalene, fluorene, acenaphthene, dibenzofuran, dibenzothiophene, phenanthrene, and anthracene (21).

Previously, based on metabolic, genomic, and transcriptomic data, we described the pyrene degradation pathway in *Cycloclasticus* sp. strain P1 from pyrene to the central TCA cycle intermediates; this pathway converges with the degradation pathways of both naphthalene and phenanthrene (19). In strain P1, pyrene degradation begins when pyrene dioxygenase (RHD-1) attacks the aromatic ring to form pyrene-*cis*-4,5-dihydrodiol, which is subsequently dehydrogenated to 4,5-dihydroxypyrene by a dihydrodiol dehydrogenase. Phenanthrene dioxygenase (RHD-2) catabolizes phenanthrene via an initial dioxygenation at the bay region, producing phenanthrene-*cis*-3,4-dihydrodiol (19). Phenanthrene-*cis*-3,4-dihydrodiol is then dehydrogenated to 3,4-dihydroxyphenanthrene by a dihydrodiol dehydrogenase (19). In strain P1, the first catabolic step of naphthalene degradation is the formation of (1*R*,2*S*) naphthalene-*cis*-l,2-dihydrodiol by naphthalene 1,2-dioxygenase (RHD-3); (1*R*,2*S*) naphthalene-*cis*-l,2-dihydrodiol is then dehydrogenated to 1,2-dihydroxynaphthalene by an NAD-dependent *cis*-1,2-naphthalenedihydrodiol dehydrogenase (19). It is intriguing that this bacterium initiates distinct, specialized pathways for pyrene, phenanthrene, and naphthalene degradation.

We recently identified two regulator genes, *dpr-1* (Q91_0868) and *dpr-2* (Q91_2232), in the gene clusters of the PAH degradation pathways of *Cycloclasticus* sp. strain P1 (19). *dpr-1* and *dpr-2* encode regulatory proteins in the CRP/FNR family (here named DPR-1 and DPR-2, respectively). Although both proteins were upregulated in the presence of naphthalene, phenanthrene, or pyrene (19), their specific responses to these PAHs differed. However, it remains unclear how cells perceive intracellular PAHs and how cells transmit the PAH signal to different regulators.

To understand the PAH-specific signal transduction pathway, we screened key genes involved in PAH utilization from a random mutation library for *Cycloclasticus* sp. strain P1 and characterized the key genes essential for PAH metabolism, in addition to those previously characterized in the PAH degradation pathways. Our results clarify the relationship between PAHs and their specific metabolic pathways, providing insights into the early responses of bacteria to PAHs. We aim to describe, for the first time, the intracellular PAH-signaling cascade; this description will increase our understanding of the intrinsic mechanisms underlying bacterial interactions with PAHs. These key data should be considered to improve the rational design and efficiency of recombinant biodegraders, bacterial biosensors, and biocatalysts in modern green chemistry.

## RESULTS

### Transposon mutant library construction in *Cycloclasticus* sp. strain P1 and screening for genes involved in PAH utilization.

To screen genes potentially involved in the PAH metabolism, a mini-Tn*5* transposon library for *Cycloclasticus* sp. strain P1 was constructed. Using a high-throughput screening method, mutant strains were identified. Of these mutant strains, 67 exhibited abnormal growth when naphthalene, phenanthrene, or pyrene was provided as the sole carbon and energy source, with acetate as the control (see Fig. S1 in the supplemental material). Corresponding interrupted genes were retrieved in these mutants by searching the genome sequence; 22 of these genes carried exact transposon insertion sites (Table 1). Notably, these interrupted genes included two CRP/FNR family transcriptional regulator genes and one diguanylate cyclase (DGC) gene (Table 1). Further complementation experiments confirmed that the deficiencies in PAH degradation observed in strains carrying mutated copies of these genes were not caused by polar effects on the genes downstream of each interrupted gene (Fig. S1).

**TABLE 1:** Genes identified as responsible for polycyclic aromatic hydrocarbon metabolic network in *Cycloclasticus* sp. strain P1 transposon mutant library

P1 mutant strain	Targeted genes in P1 (accession code)	Function or functional category	Length (aa)	Substrate(s) of mutant library screening
OMWP1	*ompW* (Q91_0761)	Outer membrane protein W	221	Nap, Phe
CSKP1	*cshk* (Q91_1119 /1120)	Sensor membrane protein	569	Nap, Phe, Pyr
CHYP1	*cheY* (Q91_1121)	*cheY*-like receiver protein	141	Nap, Phe, Pyr
OMTP1	*ompT* (Q91_2250)	Outer membrane protein transport protein	216	Pyr
DGCP1	*pdgC* (Q91_0546)	Diguanylate cyclase	445	Nap, Phe, Pyr
DPRP1-1	*dpr-1* (Q91_0868)	CRP/FNR family transcriptional regulator	242	Pyr
DPRP1-2	*dpr-2* (Q91_2232)	CRP/FNR family transcriptional regulator	238	Nap, Phe
PASP1	*pahS* (Q91_0827)	Outer membrane receptor protein	687	Nap, Phe, Pyr
PAPP1	*pahP* (Q91_0826)	Periplasmic substrate-binding protein	318	Nap, Phe, Pyr
PAKP1	*pahK* (Q91_0825)	Histidine kinase	771	Nap, Phe, Pyr
PARP1	*pahR* (Q91_1137)	Global regulator GacA	215	Nap, Phe, Pyr
RHDP1-1α	*rhd1α* (Q91_0870)	Aromatic-ring-hydroxylating dioxygenase, alpha subunit	443	Pyr
RHDP1-1β	*rhd1β* (Q91_0871)	Aromatic-ring-hydroxylating dioxygenase, beta subunit	174	Pyr
RHDP1-1α	*rhd2α* (Q91_2244)	Aromatic-ring-hydroxylating dioxygenase, alpha subunit	443	Phe
RHDP1-3α	*rhd3α* (Q91_2225)	Aromatic-ring-hydroxylating dioxygenase, alpha subunit	177	Nap
FERP1	*fer* (Q91_2219)	PAH dioxygenase component ferredoxin	104	Nap, Phe, Pyr
FRRP1	*ferR* (Q91_2220)	PAH dioxygenase component ferredoxin reductase	340	Nap, Phe, Pyr
PETP1-1	*perT1* (Q91_2242)	Permease protein	302	Nap, Phe
PETP1-2	*perT2* (Q91_0869)	Permease protein	534	Pyr
REDP1	*rcd* (Q91_2224)	Ring cleavage dioxygenase	298	Nap, Phe, Pyr
CSAP1	*csrA* (Q91_0863)	Carbon storage regulator	61	Nap, Phe, Pyr
XYEP1	*xylE* (Q91_0505)	Catechol 2,3 dioxygenase, XylE	306	Nap, Phe, Pyr

10.1128/mSystems.00636-21.1 FIG S1 Complementation of gene activity in the interrupted gene mutant strains with the corresponding genes expressed from the mini-Tn*7* site-specific transposition plasmid (see Tables S1 and S5 at https://doi.org/10.6084/m9.figshare.15042720.v1). The data show the growth of the corresponding complementation mutant strains in 100 ml ASM medium supplemented with 0.1% (wt/vol) naphthalene, phenanthrene, and pyrene. Growth was measured as the increase in OD value in cultures over time. Data represent the means from three independent experiments. Error bars represent the SD. Download FIG S1, PDF file, 1.7 MB. Copyright © 2021 Wang and Shao. 2021 Wang and Shao. https://creativecommons.org/licenses/by/4.0/ This content is distributed under the terms of the Creative Commons Attribution 4.0 International license.

### Intracellular PAH signal sensing and transduction occurs via c-di-GMP.

**FIG 1:**
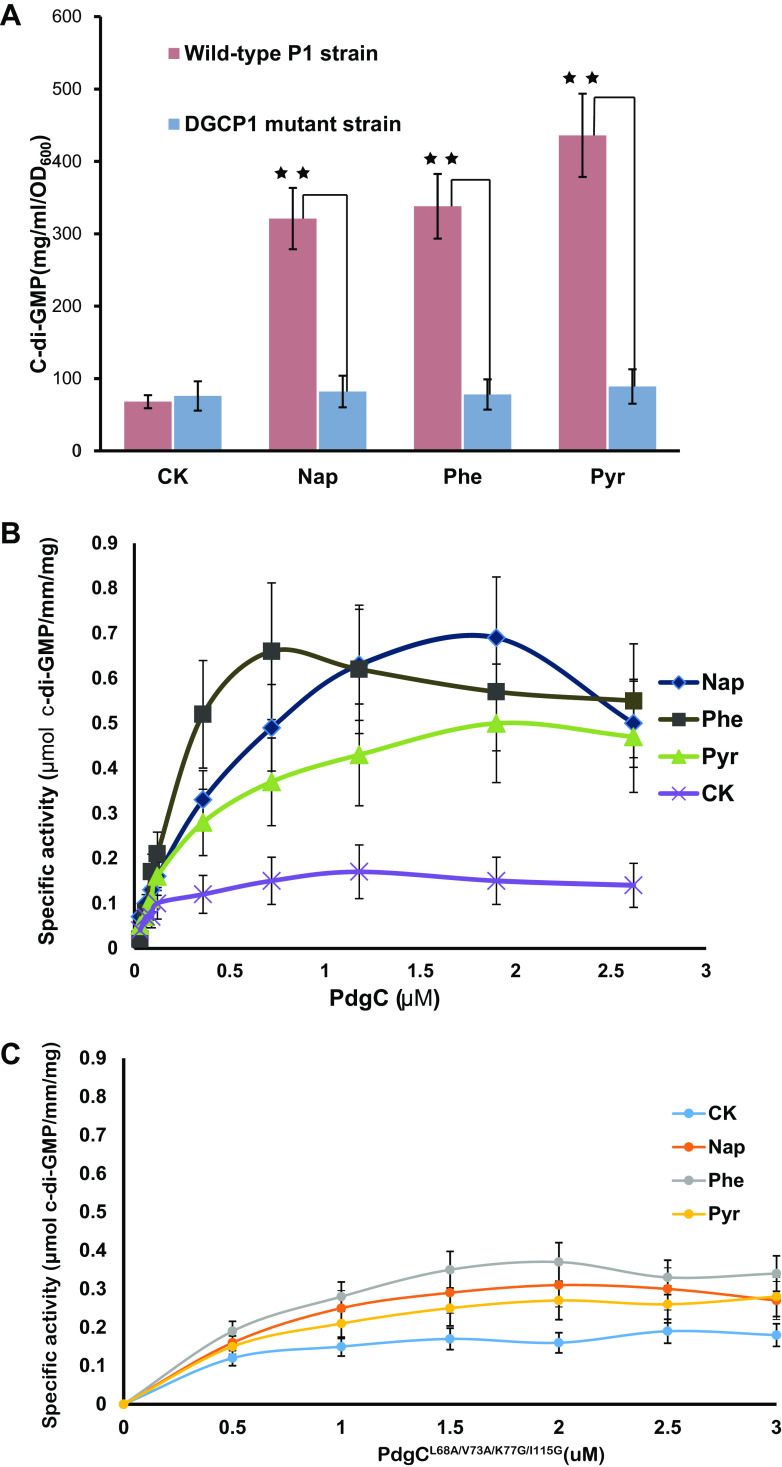
Functional analysis of PdgC proteins. (A) Cyclic di-GMP concentrations in samples of wild-type *Cycloclasticus* sp. strain P1 and DGCP1 mutant cultures treated with naphthalene, phenanthrene, or pyrene. Strain P1 cells, obtained from OD-adjusted overnight precultures, were inoculated onto various PAH substrates and grown for 48 h in triplicate. (B and C) Specific activity of PdgC (B) and PdgC^L68A/V73A/K77G/I115G^ (C) in response to different PAHs. The PdgC or PdgC^L68A/V73A/K77G/I115G^ protein was equilibrated in assay buffer with or without binding to a PAH (naphthalene, phenanthrene, or pyrene was dissolved in DMSO to a final concentration of 2 μM), and enzymatic activity was assayed at PdgC or PdgC^L68A/V73A/K77G/I115G^ concentrations of 0.05 to 3.0 μM. The values shown are averages from three independent replicates. The error bars represent standard deviations (SD). Abbreviations: Nap, naphthalene; Phe, phenanthrene; Pyr, pyrene; and CK, control group (not treated with PAHs). **, highly significant (*P* < 0.01). For details, see Materials and Methods.

Upon library screening, we noticed that one of the mutant strains (here named DCGP1) exhibited significantly reduced growth compared to the wild-type strain when PAHs were used as the sole carbon source. In strain DCGP1, we precisely identified the transposon insertion site in gene Q91_0546, here named *pdgC*. We tentatively predicted that *pdgC* encoded a DGC that catalyzes GTP to generate the small secondary messenger molecule c-di-GMP. Previously, we found that *pdgC* was upregulated 6.5- to 8.0-fold in the presence of pyrene, phenanthrene, or naphthalene (19). Here, the intracellular concentration of c-di-GMP was 8- to 10-fold greater in wild-type strain P1 than the PAH-absent control after exposure to pyrene, phenanthrene, or naphthalene. Pyrene exposure led to the highest level of c-di-GMP production, 30 to 35% higher than that caused by naphthalene or phenanthrene (Fig. 1A). However, in the PdgC mutant strain, c-di-GMP did not increase in the presence of any tested PAH (Fig. 1A).

**FIG 2:**
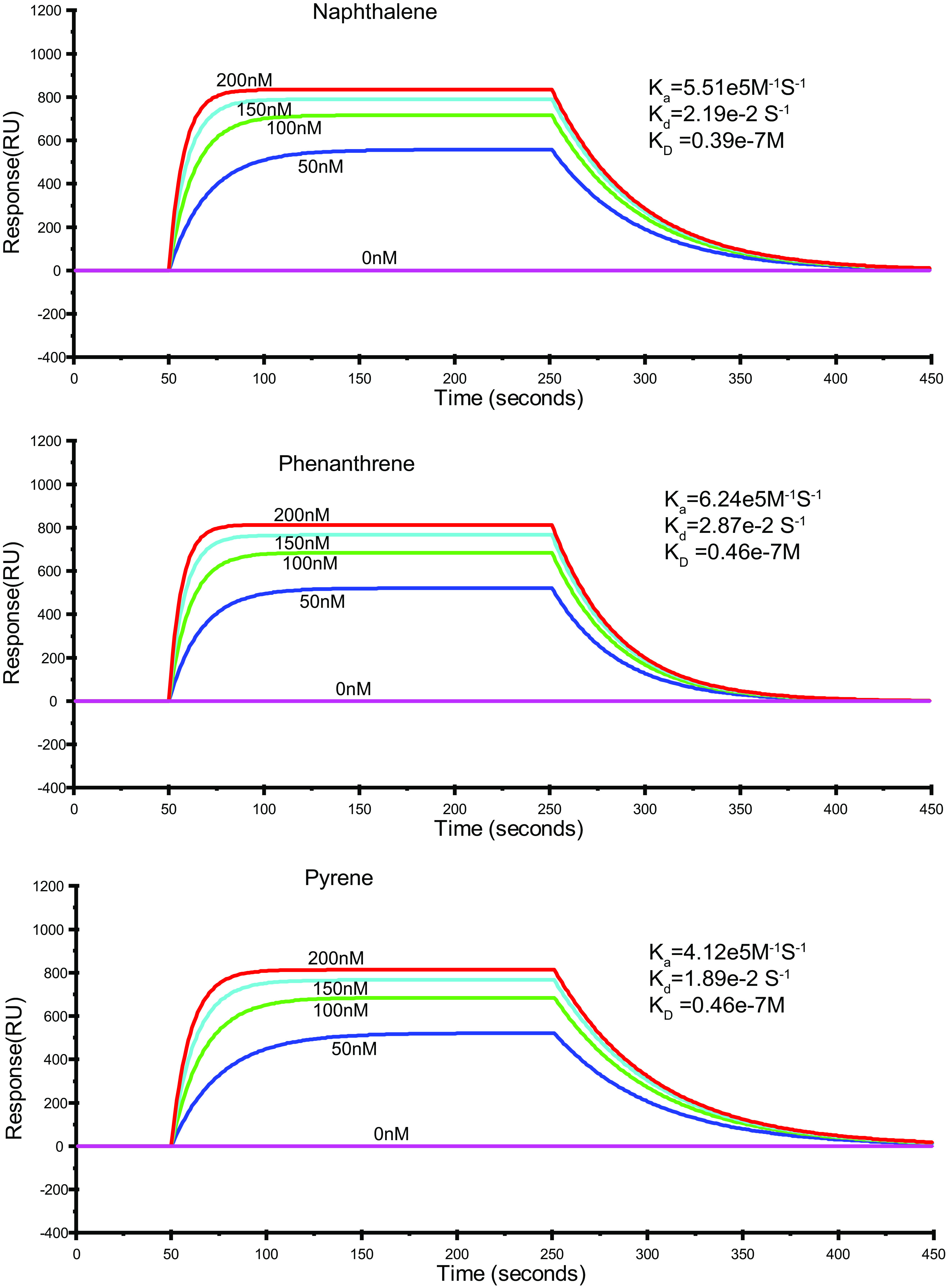
Surface plasmon resonance (SPR) measurement of affinity constants between PdgC and various PAHs. Representative SPR sensograms, recorded after injection of different concentrations of naphthalene, phenanthrene, or pyrene into a PdgC sensor chip. The values of the affinity constant (*K_D_*) were calculated by global fitting using a steady-state affinity model. Data were representative of three independent experiments.

To investigate the regulatory processes associated with c-di-GMP, we first assessed the binding potentials of various PAHs to PdgC. PdgC was expressed in Escherichia coli and purified. The binding potentials of various PAHs then were measured using surface plasmon resonance (SPR) assays. We found that PdgC bound to all tested PAHs. At 20°C, the affinity constant (*K_D_*) values calculated for PdgC with the PAH cofactor were 0.39 × 10^−7^ M, 0.46 × 10^−7^ M, and 0.46 × 10^−7^ M for naphthalene, phenanthrene, and pyrene, respectively (Fig. 2).

To assess the specificity of the response of PdgC to various PAHs, PdgC activity was measured *in vitro* with inorganic pyrophosphate assays using Enzchek pyrophosphate assay kits (as described in Materials and Methods). The results showed that PdgC was active only in the presence of PAHs such as naphthalene, phenanthrene, and pyrene (Fig. 1B). Thus, PAHs stimulate PdgC to generate c-di-GMP. This is the first report that PAHs can function as effectors of DGC during the enzymatic activity leading to c-di-GMP generation. To gain insights into the intrinsic mechanisms of PAH signal transduction, we analyzed the DGC sequence. We found that PdgC possessed two superdomains, one potential sensor domain (here named Per-Arnt-Sim, or PAS) and one catalytic domain, with the signature sequence GG(D/E)EF, that generated c-di-GMP (Fig. S2A). However, PdgC lacked transmembrane regions, indicating that it is located in the cytoplasm.

10.1128/mSystems.00636-21.2 FIG S2 Genomic map showing the domain organization of PdgC (A) and DPR (B) using the Simple Modular Architecture Research Tool (SMART; https://smart.embl.de). PAS, Per-Arnt-Sim sensor domain; GGDEF, GG(D/E)EF domain; cNMP, cyclic nucleotide (cNMP)-binding domain; HTH, helix-turn-helix (HTH) DNA-binding domain. Download FIG S2, PDF file, 0.2 MB. Copyright © 2021 Wang and Shao. 2021 Wang and Shao. https://creativecommons.org/licenses/by/4.0/ This content is distributed under the terms of the Creative Commons Attribution 4.0 International license.

**FIG 3:**
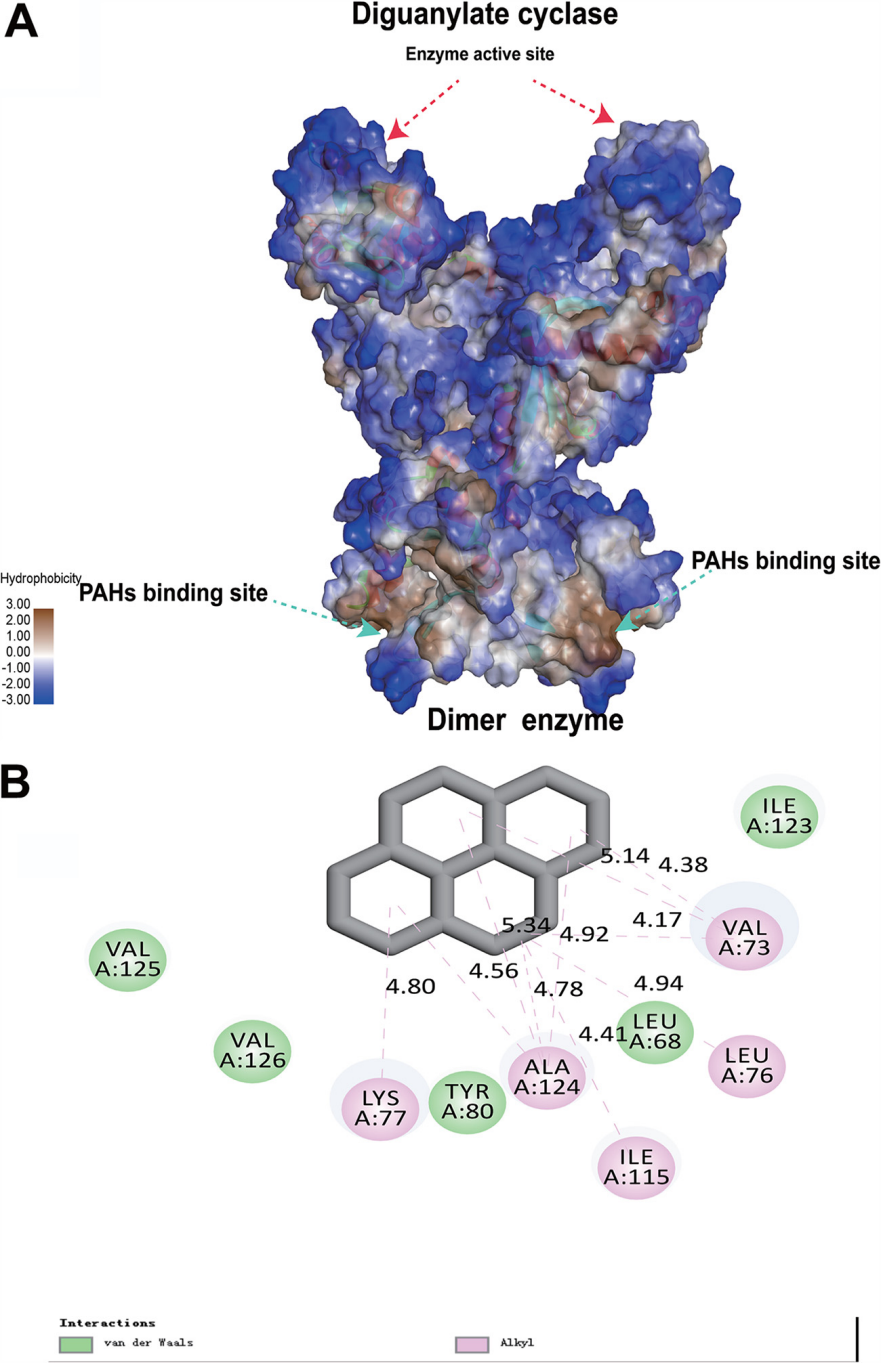
Modeling of PAH interactions with PdgC using Discovery Studio 2019. (A) Three-dimensional (3D) models of the interactions between DGC and pyrene. (B) 2D models of the interactions between PdgC and pyrene.

Homology modeling using Discovery Studio 2019 (22) predicted that PdgC was a closely packed dimer (Fig. 3A). The core structure of the PAS domain was composed of a six-stranded antiparallel β-sheet augmented by short α-helices; this topological arrangement led to the formation of a barrel-shaped module, housing a hydrophobic pocket, in the interior of the PAS domain (Fig. 3A). Analysis of the binding models using Discovery Studio 2019 showed that five residues with aliphatic side chains (Leu68, Val73, Lys77, Ile115, and Ala124) formed a cavity (Fig. 3B).

To confirm this binding model, we performed substitution mutations in four of the aliphatic amino acids: Leu68-Ala68, Val73-Ala73, Lys77-Gly77, and Ile115-Gly115. The binding ability of PdgC^L68A/V73A/K77G/I115G^ mutant to each PAH (naphthalene, phenanthrene, or pyrene) was examined using SPR. The resulting *K_D_* values, all of which were about 10^−4^ M, indicated that these site mutations decreased the binding capacity of PdgC to PAHs (Fig. S3). These *K_D_* values were significantly greater than those measured for wild-type PdgC (about 10^−7^ M), indicating that the PAS domain of PdgC was responsible for PAH binding and recognition. However, in the PdgC^L68A/V73A/K77G/I115G^ mutant activity *in vitro*, c-di-GMP did not increase in the presence of any tested PAH compared with the control group (Fig. 1C). At the C terminus of the PAS domain, two α-helical segments projected away from the core of the PAS domain and connected with the GGDEF domain (Fig. 3A). The theoretical structure of the GGDEF domain primarily consisted of four α-helices and four β-strands, with anti-parallel beta strands wrapped around the alpha helices (Fig. 3A).

10.1128/mSystems.00636-21.3 FIG S3 Measurement of affinity constant by SPR analysis. Representative SPR sensograms recorded after injection of different concentrations of naphthalene, phenanthrene, and pyrene in a four-site mutation PdgC (Leu68–Ala68, Val73–Ala73, Lys77–Gly77, and Ile115–Gly115) sensor chip. The affinity constant *K_D_* values were calculated by global fitting using a steady-state affinity model. Data were representatives of three independent experiments. Download FIG S3, PDF file, 0.4 MB. Copyright © 2021 Wang and Shao. 2021 Wang and Shao. https://creativecommons.org/licenses/by/4.0/ This content is distributed under the terms of the Creative Commons Attribution 4.0 International license.

### Characterization of two transcriptional regulators of PAH degradation pathways.

The first regulator, DPR-1, which is encoded by the gene Q91_0868, was located upstream of the pyrene-degrading dioxygenase gene *rhd1*. In wild-type strain P1, DPR-1 was significantly upregulated in the presence of pyrene but only slightly upregulated in the presence of naphthalene and phenanthrene (19) (Fig. S4). The disruption of gene *dpr-1* inhibited the growth of the mutant DPRP1-1 on pyrene but not on naphthalene and phenanthrene (Fig. S1). This indicated that DPR-1 is a positive regulator of the pyrene degradation pathway (described in detail below).

10.1128/mSystems.00636-21.4 FIG S4 Expression of *dpr-1* and *dpr-2* in wild-type P1. The wild-type P1 strains were grown on PAHs carbon sources; control cells were cultured on sodium acetate. The data are presented as the mean of three independent experiments. The error bars represent the SD. Abbreviations: Nap, naphthalene; Phe, phenanthrene; Pyr, pyrene; and CK, control group (non-PAH treatment). **, highly significant (*P* < 0.0l). For details, see the Materials and Methods. Download FIG S4, PDF file, 0.3 MB. Copyright © 2021 Wang and Shao. 2021 Wang and Shao. https://creativecommons.org/licenses/by/4.0/ This content is distributed under the terms of the Creative Commons Attribution 4.0 International license.

The other regulator, DPR-2, encoded by the gene Q91_2232, was located in the cluster of naphthalene/phenanthrene-degrading genes (19). In contrast to DPR-1, DPR-2 was slightly upregulated under naphthalene or phenanthrene treatment in the wild-type P1 strain (Fig. S4). The mutation of *dpr-2* generated the mutated strain DPRP1-2 (△*dpr-2¯*). This strain did not grow on naphthalene or phenanthrene but grew normally on pyrene (Fig. S1). This indicated that DPR-2 is a positive regulator specific to the naphthalene/phenanthrene degradation pathway.

Protein sequence analysis showed that both DPR-1 and DPR-2 possess two domains: the C-terminal domain, which contains a characteristic helix-turn-helix (HTH) motif and which mediates interactions with target DNA sequences, and the N-terminal domain, which contains a cyclic nucleotide (cNMP)-binding motif that binds cNMP and activates the C-terminal DNA-binding domain (Fig. S2B).

### c-di-GMP was the effector of the two DPR transcriptional regulators.

**FIG 4:**
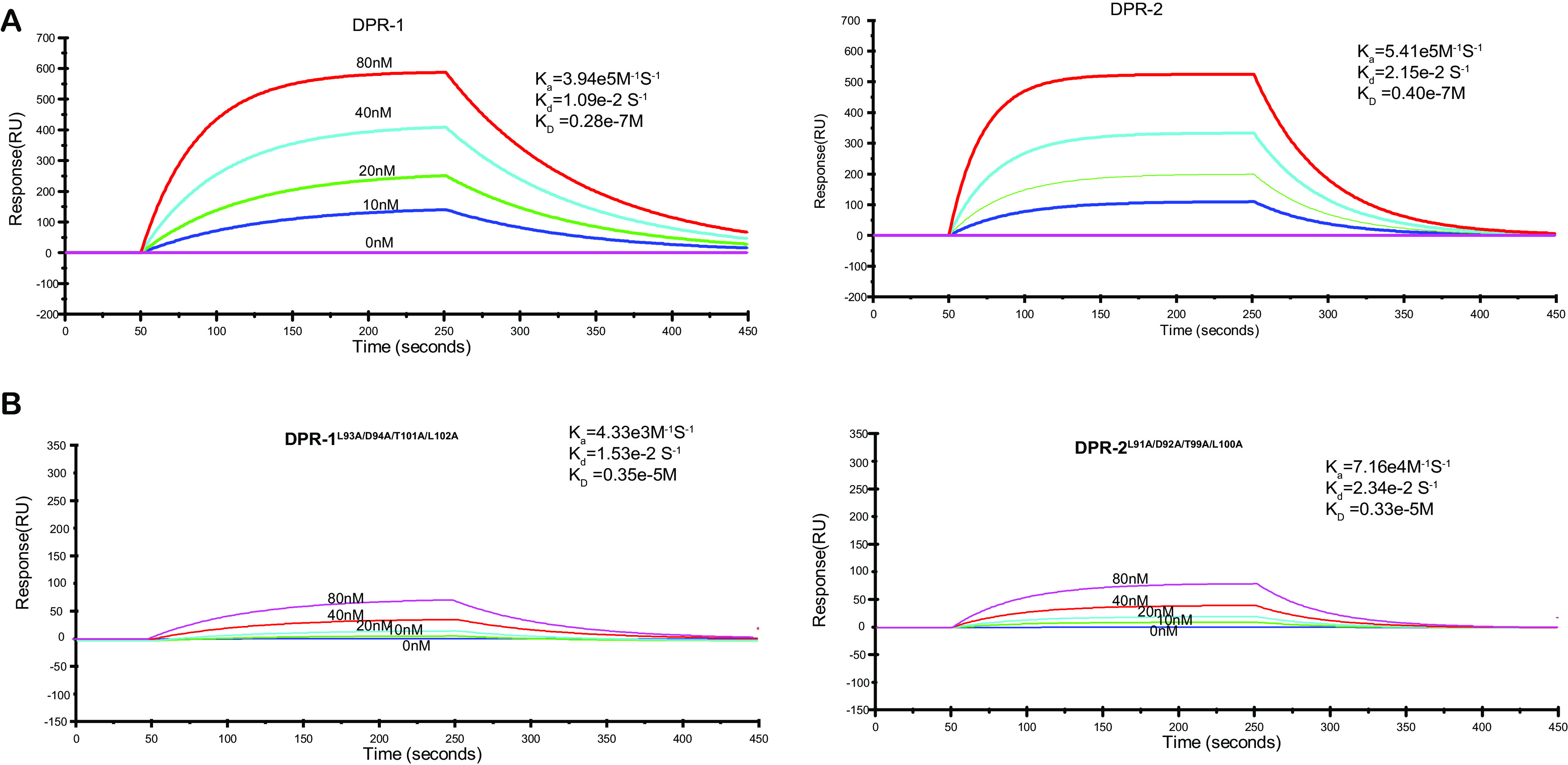
SPR examination of the ligand profiles of DPRs. SPR data for the binding of c-di-GMP to DPR-1 or DPR-2 (A) and DPR-1^L93A/D94A/T101A/L102A^ or DPR-2^L91A/D92A/T99A/L100A^ (B). Representative SPR sensograms, recorded after injection of different concentrations of c-di-GMP into the DPR-1/2 or DPR-1^L93A/D94A/T101A/L102A^/DPR-2^L91A/D92A/T99A/L100A^ sensor chip, were used. The values of the affinity constant (*K_D_*) were calculated by global fitting, using a steady-state affinity model. Data were representatives of three independent experiments.

As the catalytic product of PdgC ligated with PAHs, c-di-GMP must play a key role in degradation pathway regulation via the PAH signal relay. The interactions between DPR and secondary message molecules, including c-di-GMP, c-di-AMP, and cyclic AMP (cAMP), were examined using SPR assays. The results showed that both DPR-1 and DPR-2 used c-di-GMP as an effector rather than cAMP or c-di-AMP (Fig. S7). At 20°C, the binding of c-di-GMP to DPR-1 had a *K_D_* value of 0.28 × 10^−7^ M, while that of c-di-GMP to DPR-2 had a *K_D_* value 0.40 × 10^−7^ M (Fig. 4A).

10.1128/mSystems.00636-21.7 FIG S7 Measurement of affinity constant by SPR analysis. SPR data for the binding of cAMP and c-di-AMP to DPR1 and DPR2, respectively. Representative SPR sensograms, recorded after injection of different concentrations of cAMP or c-di-AMP into the DPR-1 or DPR-2 sensor chip. The values of the affinity constant (*K_D_*) were calculated by global fitting, using a steady-state affinity model. Data were representative of three independent experiments. Download FIG S7, PDF file, 0.4 MB. Copyright © 2021 Wang and Shao. 2021 Wang and Shao. https://creativecommons.org/licenses/by/4.0/ This content is distributed under the terms of the Creative Commons Attribution 4.0 International license.

**FIG 5:**
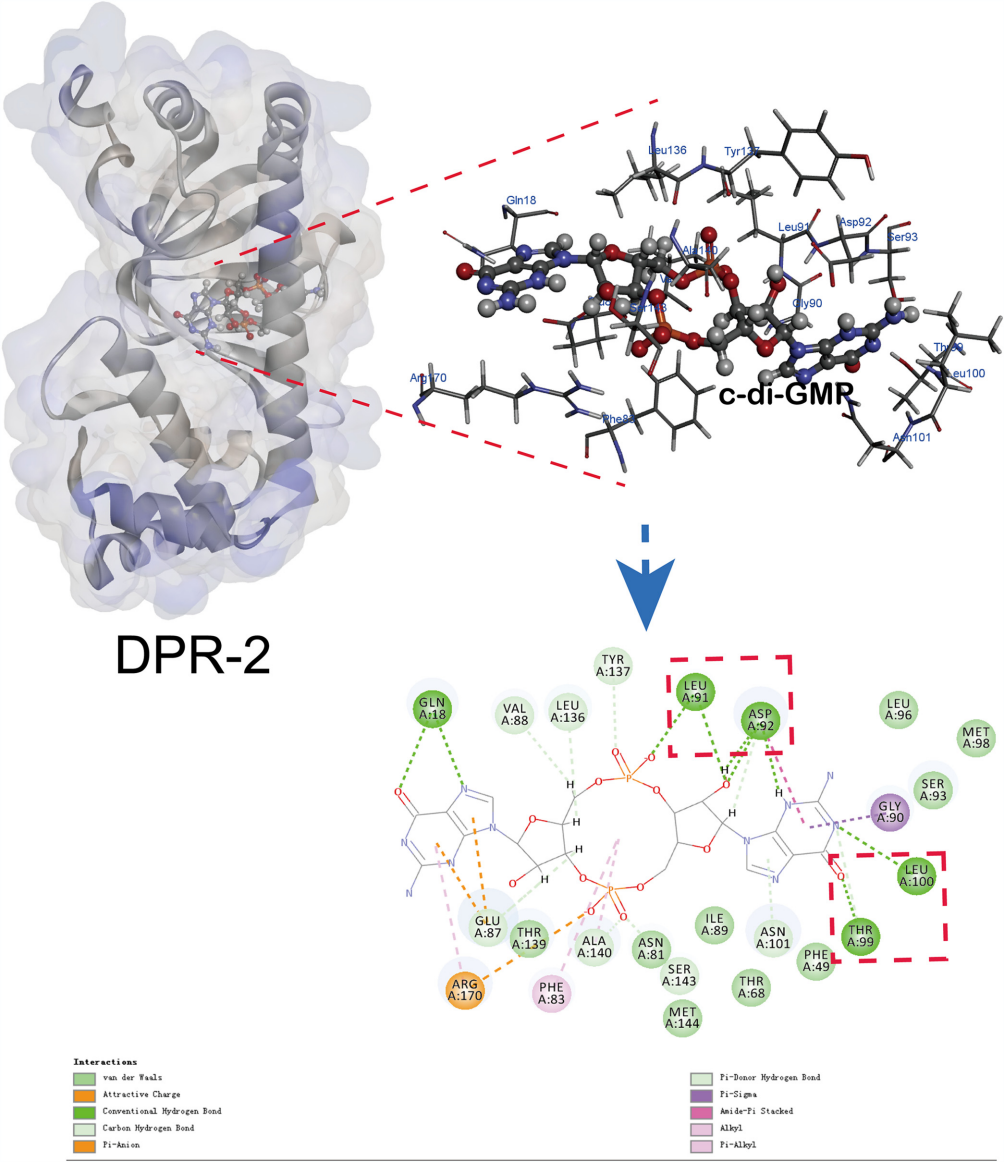
Modeling of c-di-GMP interaction with DPR-2 by Discovery Studio 2019. Both 3D (upper) and 2D (lower) models of DPR-2 interactions with c-di-GMP pyrene are depicted. The red box represents the amino acid sites with hydrogen bonding ability.

To determine the mechanism of c-di-GMP binding, the theoretical structure of DPR-1 or DPR-2 was solved through homology modeling (templates 5e44.1.A and 5cvr.1.A), and docked models were established with Discovery Studio 2019 (see Materials and Methods). The docked models are depicted in Fig. 5. In the case of DPR-2, most of the docked c-di-GMP models turned out to be residing in a local hydrophobic cavity between the cNMP domain and HTH domain (Fig. 5). The c-di-GMP molecule is accommodated well into DPR-2 via four conventional hydrogen bonds between the guanine link ring or guanine base and Leu91 (between the side chain methyl group of L91 and the O atoms of phosphate group of link ring and O atoms of guanine base), Asp92 (between the side chain carboxylate of D92 and the N and O atoms of guanine base), Thr99 (between the side chain methyl group of T99 and the O atom of guanine base), and Leu100 (between the side chain methyl group of L100 and the N atoms of guanine base) (Fig. 5). The interactions between c-di-GMP, DPR-1, and the docked models were also established with Discovery Studio 2019 (see Materials and Methods). The duplicate binding models are depicted in Fig. S8.

10.1128/mSystems.00636-21.8 FIG S8 Modeling of predict the amino acids necessary for DPR-1 or DPR-2 to bind to c-di-GMP using Discovery Studio 2019. The superimposed results of DPR-1 and DPR-2 are shown in the upper half. Dpr-1 and DPR-2 are colored grey and blue, respectively. Sequence alignment results of DPR-1 and DPR-2 are shown in the lower half. Download FIG S8, TIF file, 2.3 MB. Copyright © 2021 Wang and Shao. 2021 Wang and Shao. https://creativecommons.org/licenses/by/4.0/ This content is distributed under the terms of the Creative Commons Attribution 4.0 International license.

To reconfirm this binding model, site mutations were introduced into DPR-2 (or DPR-1) at L91/A91, D92/A92, T99/A99, and L100/A100, resulting in a quadruple mutated protein, DPR-2^L91A/D92A/T99A/L100A^ (or DPR-1^L93A/D94A/T101A/L102A^). The substrate-binding ability of the mutants then was examined using SPR to c-di-GMP. The *K_D_* value was about 10^−5^ M (Fig. 4B), which was significantly higher than that of wild-type DPR (about 10^−7^ M). These results confirmed that c-di-GMP is a secondary messenger that acts as an effector of two pathway regulators to modulate PAH metabolism in strain P1.

### Genes regulated by DPR1 and DPR2, as revealed by ChIP-seq and confirmed using SPR.

To identify genes under the regulatory control of the DPR regulators, DPR-targeted DNA-binding sequences that potentially function as promoters of genes involved in the PAH metabolism were retrieved from the genome using chromatin immunoprecipitation sequencing (ChIP-seq) (as described in Materials and Methods). Integrated analyses of the short fragments in the genomic and ChIP-seq data revealed that most of the binding sites were located in the intergenic spacer regions. We identified several genes possibly regulated by DPR-1 or DPR-2 (see Tables S1 and S2, respectively, at https://doi.org/10.6084/m9.figshare.15042720.v1).

**FIG 6:**
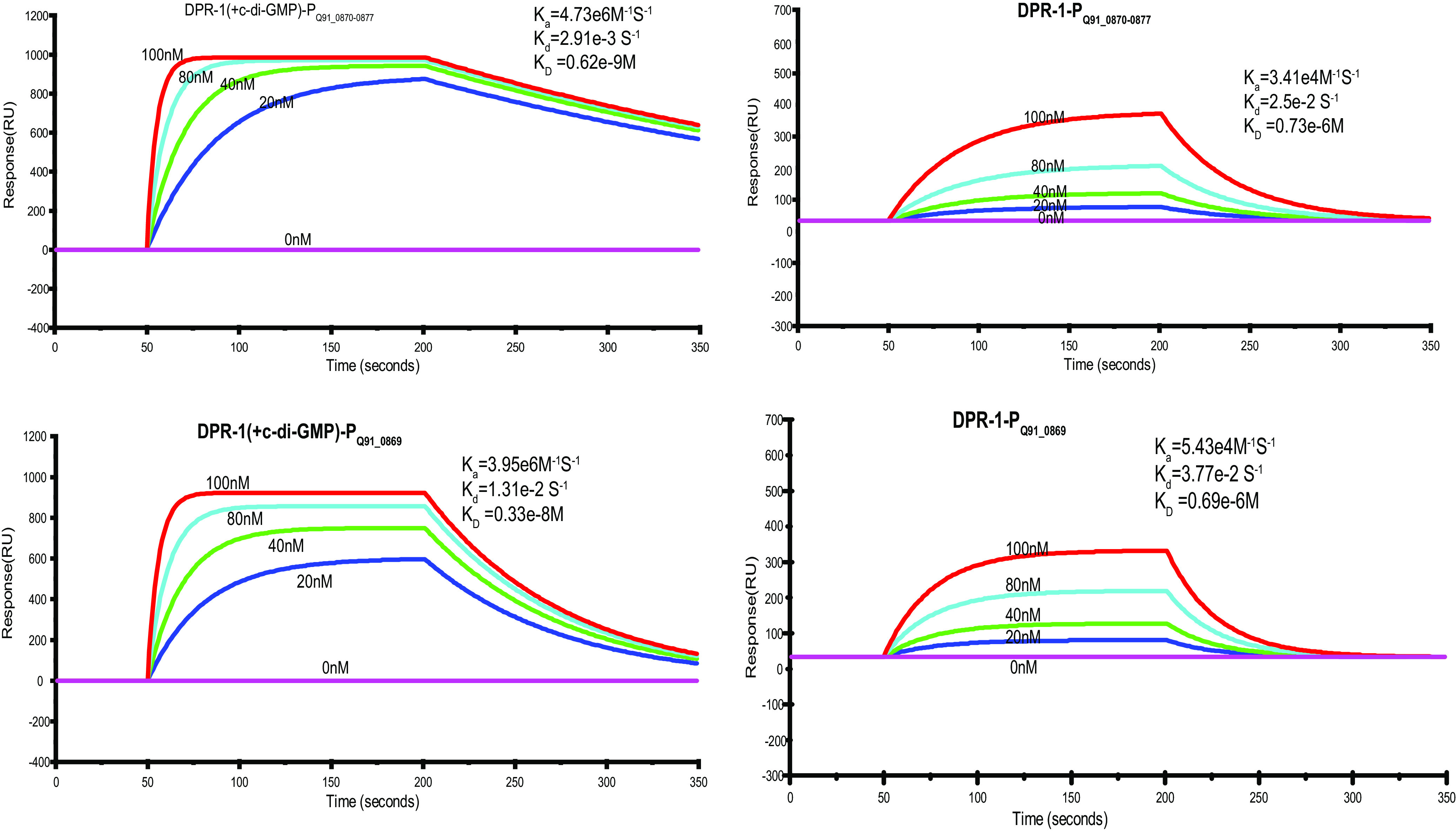
Quantification of DPR-1 DNA binding using SPR spectroscopy. The biotin-labeled DNA fragments P*_Q91_0870-0877_* and P*_Q91_0869_* were captured on a streptavidin-coated sensor chip, and purified DPR-1 or DPR-1 bound to c-di-GMP (80 nM) was passed over the chip at a flow rate of 30 μl/min at 25°C (DPR-1 concentrations were 0, 20, 40, 80, and 100 nM). The contact (association) time was 150 s, followed by a 150-s dissociation phase. Increasing RU was correlated with the rising DPR-1 concentration. Below the sensorgrams, quantifications of the binding kinetics are shown, assuming 1:1 binding. Association rates (*K_a_*), dissociation rates (*K_d_*), and overall affinities (*K_D_*) are shown.

To identify genes targeted by DPR-1 using ChIP-seq, 3,728 fragments were sequenced. Analysis revealed that DPR-1 targeted only two sequence sites in the genomic DNA (gDNA; Table S1 at https://doi.org/10.6084/m9.figshare.15042720.v1). Of the 3,728 fragments, 2,795 focused on the same site (Q91_0870-0877) (Table S1 at https://doi.org/10.6084/m9.figshare.15042720.v1). Q91_0870-0877 is the potential promoter of an operon containing several pyrene-degrading related genes, including *rhd*-*1* and *rhd*-*4*, which encode PAH dioxygenase RHD-1 and dioxygenase RHD-4, respectively (19). The promoter of a permease gene, *perT*2 (Q91_0869), was also among regulatory targets. Disruption of the regulatory gene *dpr*-*1* reduced the expression of several genes associated with pyrene degradation (Q91_0870/0871 PAH dioxygenase gene *rhd*-*1*, Q91_0872 dehydrogenase gene, Q91_0873 iron-sulfur ferredoxin gene, Q91_0874 hypothetical protein gene, Q91_0875/0876 dioxygenase gene *rhd*-*4*, and Q91_ 0877 dehydrogenase gene) as well as the permease gene *perT*2. The resulting mutant strain did not respond to pyrene (Fig. S5). This demonstrated that DPR-1 regulates the expression of pyrene-degrading genes. In addition, SPR assays revealed that, in the presence of c-di-GMP, DPR-1 displayed the highest affinity with the two promoters of pyrene-degrading genes: an operon (P_Q91_0870-0877_; *K_D_* of 0.62 × 10^−9^ M) and *perT*2 (P_Q91_0869_; *K_D_* of 0.33 × 10^−8^ M) (Fig. 6A). Without c-di-GMP, the affinities of DPR-1 for the operon and *perT2* were 500 to 100 times lower (*K_D_* values of 0.73 × 10^6^ M and 0.69 × 10^−6^ M, respectively) (Fig. 6). Interestingly, whether c-di-GMP exists or not, mutated protein DPR-1^L93A/D94A/T101A/L102A^ displayed low affinity with these two promoters of pyrene-degrading genes (Fig. S9). This again showed that regulator DPR-1 only bound the promoters of genes associated with pyrene degradation in the presence of c-di-GMP.

10.1128/mSystems.00636-21.5 FIG S5 Expression of *Q91_0869-Q91_0877* in wild-type P1 (A), *dpr-1*-deficient mutant DPRP1-1 (B), and *pdgC*-deficient mutant DGCP1 (C). The wild-type P1, DPRP1-1, and DGCP1 strains were grown on PAHs carbon sources; control cells were cultured on sodium acetate. The data are presented as the means from three independent experiments. The error bars represent the SD. Abbreviations: Pyr, pyrene; and CK, control group (non-PAH treatment). **, highly significant (*P* < 0.0l). For details, see Materials and Methods. Download FIG S5, PDF file, 1.3 MB. Copyright © 2021 Wang and Shao. 2021 Wang and Shao. https://creativecommons.org/licenses/by/4.0/ This content is distributed under the terms of the Creative Commons Attribution 4.0 International license.

10.1128/mSystems.00636-21.9 FIG S9 Quantification of DPR-1^L93A/D94A/T101A/L102A^ DNA binding using SPR spectroscopy. The biotin-labeled DNA fragments P*_Q91_0870-0877_* and P*_Q91_0869_* were captured onto a streptavidin-coated sensor chip, and purified DPR-1^L93A/D94A/T101A/L102A^ or DPR-1^L93A/D94A/T101A/L102A^ bound to c-di-GMP (80 nM) was passed over the chip at a flow rate of 30 μl/min at 25°C (DPR-1 concentrations were 0, 20, 40, 80, and 100 nM). The contact (association) time was 150 s, followed by a 150-s dissociation phase. Increasing RU was correlated with the rising DPR-1 concentration. Below the sensorgrams, quantifications of the binding kinetics are shown, assuming 1:1 binding. Association rates (*K_a_*), dissociation rates (*K_d_*), and overall affinities (*K_D_*) are shown. Download FIG S9, PDF file, 0.4 MB. Copyright © 2021 Wang and Shao. 2021 Wang and Shao. https://creativecommons.org/licenses/by/4.0/ This content is distributed under the terms of the Creative Commons Attribution 4.0 International license.

DPR-2 mainly targeted three DNA sequences (ChIP-seq fragment number 3518) (Table S2 at https://doi.org/10.6084/m9.figshare.15042720.v1). Each of these sequences was a potential promoter of an operon associated with the degradation pathways of naphthalene and phenanthrene. The first operon (genes Q91_2218, Q91_2218, and Q91_2220) encoded an isomerase, PAH dioxygenase ferredoxin, and ferredoxin reductase; this electron transduction chain is shared by all PAH degradation pathways (19). The second operon (genes Q91_2224 to Q91_2227) encoded an extradiol dioxygenase, the β- and α-subunits of PAH dioxygenase RHD-3, and the large subunit of aromatic dioxygenase, respectively. RHD-3 is responsible for phenanthrene degradation (19). The third target operon (genes Q91_2242 to Q91_2244) encoded a permease and the β- and α-subunits of PAH dioxygenase RHD-2; RHD-2 has been shown to specifically function in naphthalene degradation (19). Thus, all of the targeted genes were closely associated with PAH metabolism.

**FIG 7:**
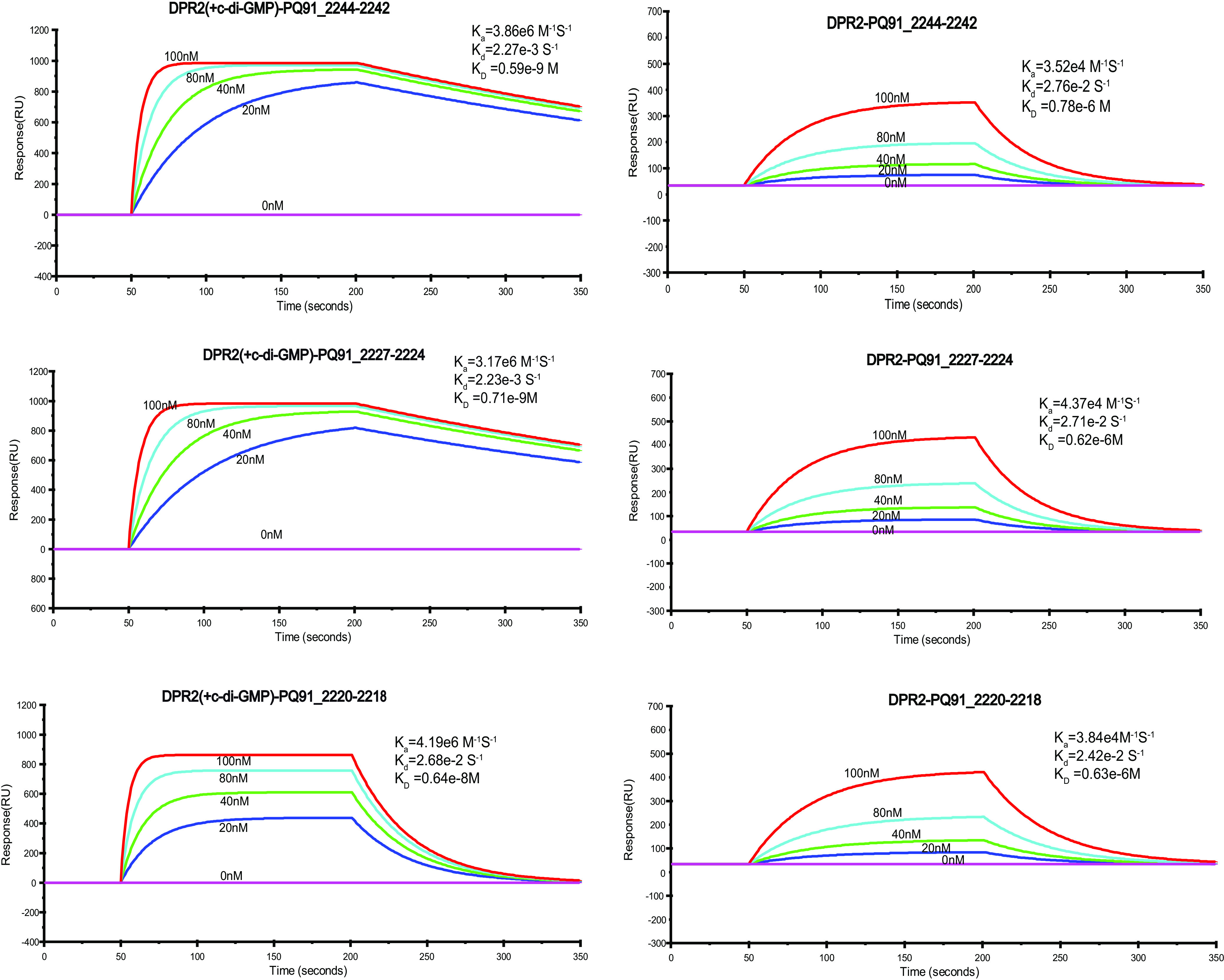
Quantification of DPR-2 DNA binding using SPR spectroscopy. The biotin-labeled DNA fragments P*_Q91_2244-2242_*, P*_Q91_2227-2224_*, and P*_Q91_2220-2218_* were captured on a streptavidin-coated sensor chip, and purified DPR-2 or DPR-2 bound to c-di-GMP (80 nM) was passed over the chip at a flow rate of 30 μl/min at 25°C (DPR-2 concentrations were 0, 20, 40, 80, and 100 nM). The contact (association) time was 150 s, followed by a 150-s dissociation phase. Increasing RU was correlated with the rising DPR-2 concentration. Below the sensorgrams, quantifications of the binding kinetics are shown, assuming 1:1 binding. Association rates (*K_a_*), dissociation rates (*K_d_*), and overall affinities (*K_D_*) are shown.

Further investigation showed that the knockout of the regulator gene *dpr*-2 eliminated the expression of each of the genes in the three operons mentioned above (Fig. S6). Similar to DPR-1, DPR-2 showed high affinity for the three promoters when bound to c-di-GMP, with *K_D_* values of 0.64 × 10^−8^ M, 0.71 × 10^−9^ M, and 0.59 × 10^−9^ M (Fig. 7). Without c-di-GMP, DPR-2 had a relatively low affinity to each of the promoters of the targeted operons, with *K_D_* values of 0.63 × 10^−6^ M, 0.62 × 10^−6^ M, and 0.78 × 10^−6^ M (Fig. 7). Whether c-di-GMP exists or not, mutated protein DPR-2^L91A/D92A/T99A/L100A^ also displayed a low affinity with these three operon promoters (see Fig. S10 at https://doi.org/10.6084/m9.figshare.15042720.v1).

10.1128/mSystems.00636-21.6 FIG S6 Expression of *Q91_2220*, *Q91_2219*, *Q91_2226*, *Q91_2225*, *Q91_2244*, *Q91_2243*, and *Q91_2242* in wild-type P1 (A), *dpr-2*-deficient mutant DPRP1-2 (B), and pdgC-deficient mutant DGCP1 (C). The wild-type P1, DPRP1-1, and DGCP1 strains were grown on PAH carbon sources; control cells were cultured on sodium acetate. The data are presented as the means from three independent experiments. The error bars represent the SD. Abbreviations: Nap, naphthalene; Phe, phenanthrene; and CK, control group (non-PAH treatment). Download FIG S6, PDF file, 1.5 MB. Copyright © 2021 Wang and Shao. 2021 Wang and Shao. https://creativecommons.org/licenses/by/4.0/ This content is distributed under the terms of the Creative Commons Attribution 4.0 International license.

### Distribution of the PAH signaling pathway is widespread in bacteria.

To determine whether similar signaling pathways were present in other PAH-degrading bacteria, we used BLAST to search for homologues of *pdgC* and *dpr* in other bacterial genomes in the public Integrated Microbial Genomes & Microbiomes (IGM/M) database. We found that 52 bacterial genomes contained homologues of all three genes. All identified homologues exhibited more than 50% sequence identity and more than 80% of each query sequence was aligned. Homologues were identified in the following genera (with the number of available genomes for each genus shown in parentheses): *Alcanivorax* (4 genomes), Acinetobacter (2 genomes), *Cycloclasticus* (21 genomes), *Halomonas* (4 genomes), Pseudomonas (7 genomes), *Erythrobacter* (1 genome), *Novosphingobium* (2 genomes), *Sphingobium* (2 genomes), *Dietzia* (3 genomes), Mycobacterium (3 genomes), *Marinobacter* (2 genomes), and *Microbacterium* (1 genome) (Table S3 at https://doi.org/10.6084/m9.figshare.15042720.v1). All strains were isolated from hydrocarbon-contaminated environments and were shown to use PAHs as a carbon source (Table S3 at https://doi.org/10.6084/m9.figshare.15042720.v1).

## DISCUSSION

**FIG 8:**
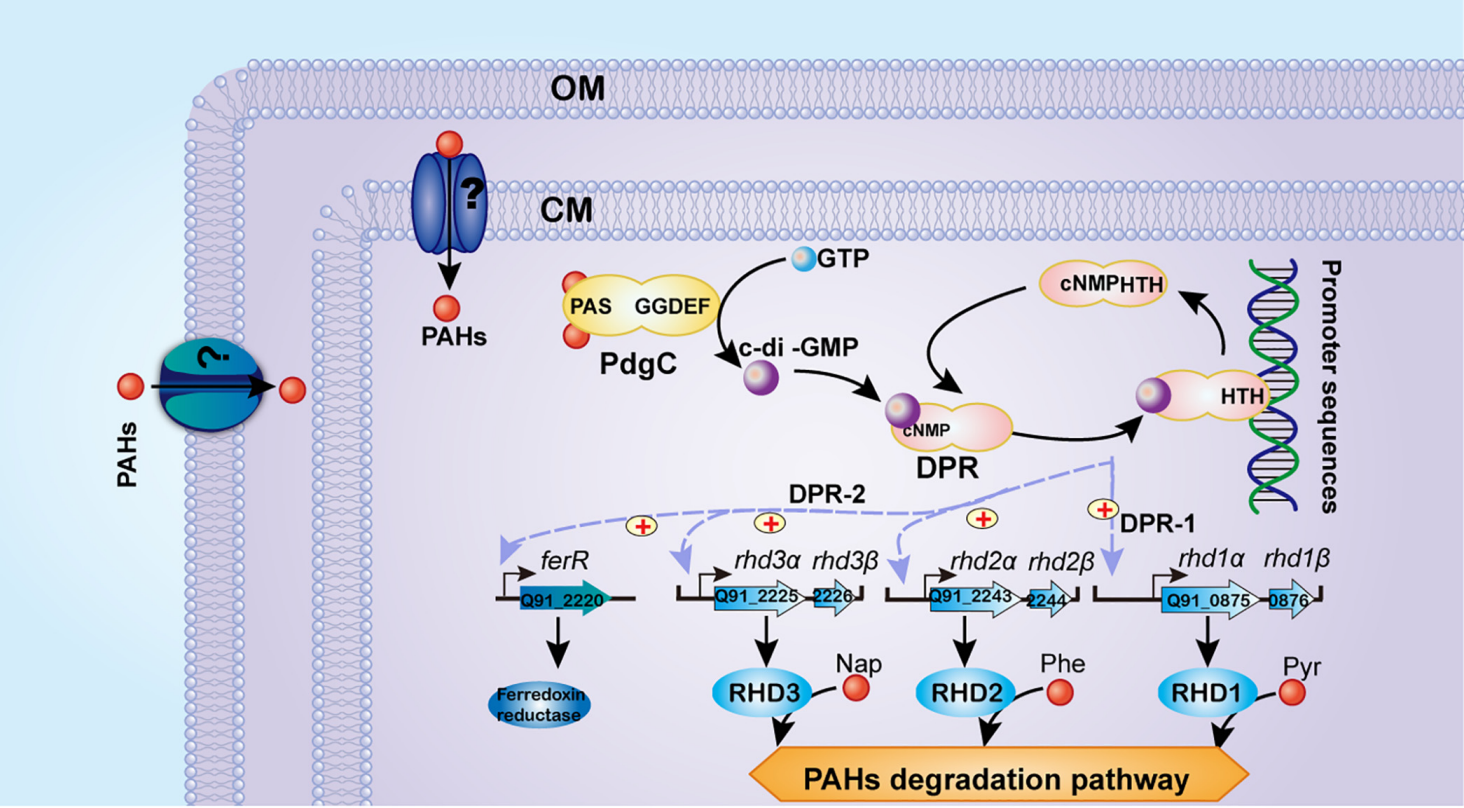
Model of the sensing system and signal transduction responses initiated by intracellular PAHs in *Cycloclasticus*. The signal-sensing and transduction pathways for PAH degradation are shown at the top or middle of the diagram (i.e., in the cytoplasm). The pathways regulating the PAH degradation pathway are shown at the bottom of the diagram. Known functional pathways are shown as solid lines; unknown or uncertain functional pathways are shown as dashed lines. A plus sign inscribed in a circle represents positive regulation.

PAH signal sensing and transduction, as well as the regulation of PAH degradation pathways, are crucial for bacterial PAH biodegradation. However, prior to this study, these processes were poorly known. Although *Cycloclasticus* bacteria are well-known as ubiquitous PAH degraders in marine environments, the mechanisms underlying the prevalence of this genus remain unclear. This study employed multiple molecular approaches to better understand the regulatory networks controlling PAH metabolism in this bacterium. This report illustrates, for the first time, PAH-specific intracellular sensing, signal transduction, and metabolic regulatory pathways. Inside the cell, PAH specifically binds to PdgC, which is specialized to sense and bind PAHs via its PAS sensor domain and which generates c-di-GMP from GTP via the GG(D/E)EF domain common in PdgC enzymes. The intracellular concentration of c-di-GMP is modulated by PdgC, which is dependent on PAHs. c-di-GMP then binds to regulators DPR-1 and DPR-2, which modulate the expression levels of a series of genes in various PAH degradation pathways (Fig. 8).

DGC triggers the synthesis of c-di-GMP (23, 24). Several DGCs with the characteristic GG(D/E)EF domain produce c-di-GMP in response to various environmental stimuli (24–26). For example, the DGC in Pseudomonas species, which is well-studied, is known to play a role in biofilm formation (27). Once activated by phosphorylation, this cyclase produces c-di-GMP, which in turn stimulates biofilm formation (28). Another well-studied DGC is PleD in Caulobacter crescentus; PleD possesses two REC domains linked to a GGDEF domain (29).

Prior to this study, little was known about DGC-associated signal transduction and the role of DGC in the hydrocarbon metabolism. Interestingly, only one DGC (PAS-GGDEF)-encoding gene in the *Cycloclasticus* sp. strain P1 genome was strongly upregulated in the presence of pyrene, phenanthrene, or naphthalene; other genes encoding potential DGC-like proteins were downregulated or not expressed in the presence of these PAHs. *In vitro* tests with the purified enzyme revealed that PdgC needed PAHs as effector molecules to generate c-di-GMP.

All tested PAHs, including naphthalene, phenanthrene, and pyrene, bound PdgC. This indicated that the PAH signal was transferred and translated into a secondary message via a PdgC enzyme specialized for PAH sensing. We assumed that the hydrolysis of c-di-GMP was not modulated by PAH, as we observed no differences in the transcription of phosphodiesterases (PDEs), which are responsible for c-di-GMP hydrolysis. Transcriptomic data showed that, regardless of PAH presence, PDEs were relatively highly expressed (19). Thus, the c-di-GMP pool in the cytoplasm is dynamic in response to PAH, and its size is mainly determined by the input of c-di-GMP via PdgC activity; PdgC expression and activity are both influenced by PAHs.

DGC protein sensory domains, possibly heme- or flavin-associated PAS domains, are in the periplasm to perceive extracellular signals associated with oxidation, redox, light, starvation, and various substances, as well as intercellular signaling molecules (24, 25). However, to the best of our knowledge, this is the first report of a DGC sensor and signal transmitter functioning in an organic-matter metabolic network.

It was noteworthy that, based on PdgC, we characterized a unique PAS domain that can bind PAHs. This is the first PAH-binding DGC PAS domain identified in prokaryotes. The PAS domain, which has been identified in all kingdoms of life, functions as a signal transducer that couples ligand binding to a cellular signaling response (30). The highly divergent PAS family is remarkably plastic, binding to a wide variety of ligands and responding to an equally wide variety of sensory stimuli (30). In prokaryotes, PAS domains are commonly positioned at the amino termini of signaling proteins, such as sensor histidine kinases, cyclic-di-GMP synthases/hydrolases, and methyl-accepting chemotaxis proteins (30). These proteins are involved in the regulation of various processes, including toluene degradation, nitrogen fixation, stress response, and virulence in pathogenic bacteria. In eukaryotic organisms, mammalian PAS domains bind to and sense endogenous or xenobiotic small molecules, including molecular oxygen and cellular metabolites.

To date, only the PAS domain of the aryl hydrocarbon receptor (AHR) has been shown to bind and sense PAHs; in mammalian cytoplasm, this receptor regulates transcriptional responses to PAHs and upregulates the cytochrome P450 family (31). Interestingly, although the PAS domain of PdgC in strain P1 was highly divergent with the PAS domain of the AHR receptor at the primary sequence level, the theoretical structures of the two PAS domains exhibited a conserved three-dimensional architecture as well as conserved binding sites (Leu68, Lys77, and Ile115 in PdgC PAS domain; see Fig. S11 at https://doi.org/10.6084/m9.figshare.15042720.v1). This implied that different life domains have similar intrinsic mechanisms to recognize PAH molecules.

As mentioned briefly above, we found that PdgC functioned as a PAH receptor in the cytoplasm, generating and transmitting signals of PAH occurrence downstream via c-di-GMP. As one of the most important secondary messengers, c-di-GMP plays a key role in signal transduction in many bacteria (25, 26, 32, 33). c-di-GMP is involved in the regulation of biofilm formation, motility, virulence, cell cycle, and cell differentiation (33–42). Most of the c-di-GMP-dependent signaling pathways identified to date involve interactions between cells and abiotic surfaces or other cells (40, 42–48). Here, for the first time, we show that c-di-GMP plays a role in a signaling network associated with an organic metabolism in bacteria. c-di-GMP connects the signaling pipeline from PAHs to the degradation pathways via PdgC and the c-di-GMP receptors.

The regulators DPR-1 and DPR-2 belong to the CRP/FNR family of proteins. CRP/FNR proteins are extraordinary in that they respond to a broad spectrum of intracellular and exogenous signals, such as cAMP, anoxia, redox state, oxidative and nitrosative stress, nitric oxide (NO), carbon monoxide (CO), 2-oxoglutarate, and temperature (49–56). Previously, regulators in this family have been associated with several processes, including the regulation of the anaerobic degradation of benzoate and cyclohexanecarboxylate in *Rhodopseudomonas palustri* (57) as well as the dehalorespiration of halogenated aromatic compounds in *Desulfitobacterium* (58). To date, transcriptional regulators in the CRP/FNR family have been identified as c-di-GMP receptors in *Xanthomonas* and *Burkholderia* bacteria only; these receptors regulate virulence gene expression and biofilm formation, respectively (59, 60). However, nothing is known about how the CRP/FNR family regulates bacterial substrate metabolisms via c-di-GMP.

In this report, we identified two c-di-GMP receptors in strain P1, DPR-1 and DPR-2, that function as master regulators of three key pathways responsible for degrading different PAHs. We then identified five operon-promoter sequences that bind to DPR-1 or DPR-2. These operons included almost all of the genes encoding PAH degradation enzymes in the previously characterized pathways. Our data also showed that both DPR regulators upregulated these genes. Interestingly, the affinity of the DPR regulators to the promoters was controlled by the PAHs via c-di-GMP. However, as the binding promoter sequences were conserved (Fig. S12 at https://doi.org/10.6084/m9.figshare.15042720.v1), the drivers of DPR-binding specificity require further investigation.

Thus, at least in *Cycloclasticus*, the PdgC-DPR-bridged regulatory system described here not only participated in the precise regulation of the cellular response to PAHs but also discriminated among different PAH substrates. This intracellular PAH signaling pathway can be used as a model of cell-PAH interactions in various organisms and may provide a useful framework for the rational design of biocatalysts and biosensors. Our results also help to clarify the evolution of PAH interactions in bacteria.

## MATERIALS AND METHODS

### Bacterial strains, chemicals, media, and culture conditions.

The strain used in this study, *Cycloclasticus* sp. strain P1 (MCCC 1A01040), was originally isolated from deep-sea sediments of the Pacific Ocean (21). Naphthalene, phenanthrene, and pyrene were purchased from Sigma-Aldrich (Shanghai, China). A 100-ml culture of strain P1 was grown in a flask in ML medium (30 g/liter NaCl, 1 g/liter NH_4_NO_3_, 0.35 g/liter KCl, 1 g/liter KH_2_PO_4_, 1 g/liter K_2_HPO_4_, 0.08 g/liter KBr, 0.05 g/liter CaCl_2_, 24 mg/liter SrCl_2_·6H_2_O, 1 × 10^−4^ g/liter ZnSO_4_·7H_2_O, 3.5 g/liter MgSO_4_·7H_2_O, 0.01 g/liter FeCl_3_, 3 g/liter sodium pyruvate, 3 g/liter sodium citrate, 0.5 g/liter yeast extract, 1 g/liter tryptone, and 1 liter of H_2_O; pH 8.0) at 28°C with shaking at 150 rpm until the optical density at 600 nm (OD_600_) reached 1.1. The culture was then divided equally among four sterile 250-ml flasks. The flasks contained 20 ppm naphthalene, phenanthrene, pyrene, or 0.5% dimethyl sulfoxide (DMSO) (control). In the PAH-containing flasks, DMSO was used as a cosolvent to normalize PAH concentrations; the final concentration of DMSO in all flasks was 0.5%. All flasks were then incubated at 28°C with shaking at 150 rpm for 7 h.

### Construction of a mini-Tn*5* transposon library.

Transposon mutagenesis was performed based on the mini-Tn*5*Km element, which was constructed as previously described (61). *Cycloclasticus* sp. strain P1 was grown in ML medium until the stationary growth phase, and the cells were then centrifuged at 3,200 × *g* at 4°C. The E. coli donor (λ pir, pUT min-Tn5 Km) and helper (pRK2013) strains were grown overnight at 37°C in LB medium supplemented with kanamycin (25 μg/ml) and then washed with fresh LB and centrifuged at 3,200 × *g* at 4°C. The pellets obtained after centrifugation (*Cycloclasticus* sp. strain P1, E. coli donor, and E. coli helper) were mixed in a 4:1:1 ratio (respectively, by volume) and placed on a membrane filter on a plate with LB agar, salts (0.45 g/liter Na_2_HPO_4_·2H_2_O, 2.5 g/liter NaNO_3_, 11.5 g/liter NaCl, 0.38 g liter^−1^ KCl, and 0.7 g/liter CaCl_2_·2H_2_O), and 2% acetate (wt vol^−1^) as the carbon and energy source. The plates were incubated for 24 h at 30°C, and cells were then washed with 10 mM MgSO_4_. Finally, transconjugants were selected on modified solid ML medium containing 30 g/liter NaCl, 1 g/liter NH_4_NO_3_, 0.35 g/liter KCl, 1 g/liter KH_2_PO_4_, 1 g/liter K_2_HPO_4_, 0.08 g/liter KBr, 0.05 g/liter CaCl_2_, 24 mg/liter SrCl_2_·6H_2_O, 1 × 10^−4^ g/liter ZnSO_4_·7H_2_O, 3.5 g/liter MgSO_4_·7H_2_O, 0.01 g/liter FeCl_3_, 3 g/liter sodium pyruvate, 3 g/liter sodium citrate, 0.5 g/liter yeast extract, 1 g/liter tryptone, 10 g/liter agar, 100 ml of strain P1 (OD_600_ of 1.1) liquid ML medium culture added after the ultrasonic crushing and filtration sterilization, and 900 ml of H_2_O (pH 7.5) supplemented with 25 μg/ml kanamycin as required.

A transposon library consisting of approximately 15,000 colonies was obtained based on the mini-Tn*5* transposon element. The *Cycloclasticus* sp. strain P1 genome contained 2,248 genes. Thus, this library represented approximately 7-fold coverage of the genome. However, mutant clones with identical disrupted genes were isolated, indicating that the library was saturated. The quality of the library was verified using Southern blot hybridization, which confirmed the randomness and uniqueness of the transposon insertions.

### High-throughput screening of the P1 transposon mutant library.

Transposon mutants from the original library were inoculated into 96-well microtiter plates containing 200 μl of artificial seawater medium (ASM) (24 g NaCl, 7.0 g MgSO_4_·7H_2_O, 1 g NH_4_NO_3_, 0.7 g KCl, 2.0 g KH_2_PO_4_, 3.0 g Na_2_HPO_4_, and 10 ml of a trace element solution at pH 7.5) supplemented with kanamycin, 0.05% (wt/vol) iodonitrotetrazolium chloride (INT), and one of naphthalene, phenanthrene, or pyrene as the sole carbon source (final concentration, 500 ppm; DMSO, 0.5% final concentration, was used as a cosolvent to normalize concentrations). The reduction of INT levels though the active respiration of growing cells stains colonies purple (62). The plates were incubated overnight at 30°C with shaking at 900 rpm. From the resulting library, we screened mutants that were unable to use PAHs (naphthalene, phenanthrene, or pyrene) as a carbon source. To confirm this initial screening and to further analyze these mutants, the selected mutant strains were replicated in 96-well microtiter plates containing ASM supplemented with kanamycin and 500 ppm of each PAH as the sole carbon source. Mutants that did not grow on the PAHs, but whose corresponding wild-type strain grew in 96-well microtiter plates supplemented with acetate, were chosen for further analysis. The mini-Tn*5* insertion sites in the selected mini-Tn*5* mutants were identified using inverse PCR (63).

### Construction of plasmids for expressing PdgC and the transcriptional regulator of DPR gene expression.

The plasmid pET-*pdgC* was constructed by amplifying *pdgC* with the DGCf/DGCr primer pair (see Table S4 at https://doi.org/10.6084/m9.figshare.15042720.v1) and cloning the resulting PCR products into the BamHI/SacI sites of pET-28a(+) (Novagen, Madison, WI, USA). The following PCR program was utilized for these assays: 95°C for 3 min, followed by 30 cycles of 95°C for 30 s, 53°C for 60 s, and 72°C for 2 min and a final extension at 72°C for 5 min. For *in vitro* experiments, pET28-*pdgC* was transfected into E. coli BL21 cells (Novagen, Madison, WI, USA). *pdgC* was then overexpressed and purified as described below.

The plasmid pET28-*dpr1* was constructed by amplifying *dpr1* with the DPR1f/DPR1r primer pair (Table S4 at https://doi.org/10.6084/m9.figshare.15042720.v1) and cloning the resulting PCR product into the BamHI/HindIII site of pET-28a(+) (Novagen, Madison, WI). Similarly, the plasmid pET28-*dpr2* was constructed by amplifying *dpr2* with the DPR2f/DPR2r primer pair (Table S4 at https://doi.org/10.6084/m9.figshare.15042720.v1) and cloning the resulting PCR product into the BamHI/SacI site of pET-28a(+) (Novagen, Madison, WI, USA). The following PCR program was utilized for these assays: 95°C for 3 min, followed by 30 cycles of 95°C for 30 s, 52°C or 55°C for 40 s, and 72°C for 2 min, and a final extension at 72°C for 5 min.

### Protein expression and purification.

To measure protein expression, the pET28-*pdgC* plasmid was transformed into E. coli BL21(DE3). Transformants were grown in 200 ml of LB broth until the cell density reached an OD_600_ of 0.6. Isopropyl beta-d-thiogalactopyranoside (IPTG) was then added to a final concentration of 1 mM. After 3 h, cells were harvested by centrifugation and washed with the lysis buffer (50 mM Tris-HCl, pH 8.0, and 100 mM NaCl) at 4°C. After the addition of phenylmethylsulfonyl fluoride (PMSF) and lysozyme (at final concentrations of 1 mM and 1 mg/ml, respectively), each cell suspension was incubated for 20 min at 4°C and then sonicated. Following treatment with RNase (10 mg/ml) and DNase (5 mg/ml) for 15 min on ice, cell extracts were obtained via centrifugation at 23,000 × *g* for 20 min at 4°C. Protein purification was performed using a Ni^2+^-chelating sepharose fast flow column (Amersham Biosciences, Piscataway, NJ) by following the procedures recommended by the manufacturer (Qiagen).

E. coli BL21(DE3) transformed with the DPR expression plasmids pET28-*dpr1* and pET28-*dpr2* (encoding His-tagged DPRs) was grown in the LB broth, and protein expression was induced by adding 1 mM IPTG. After 3 h of induction, cells were harvested and subjected to DPR purification. His-tagged DPRs were purified by following standard procedures (64, 65). In brief, lysozyme-treated cells were sonicated in the presence of 100 mM phenylmethylsulfonyl fluoride. After the cell lysate (30 ml) was centrifuged at 15,000 rpm for 60 min at 4°C, the resulting supernatant was mixed with 2 ml of 50% Ni-nitrilotriacetic acid (Ni-NTA) agarose solution (Qiagen) and loaded onto a column. After being washed with 10 ml of the lysis buffer, the column was washed with 10 ml of the washing buffer (50 mM Tris-HCl, pH 8.0, and 100 mM NaCl) at 4°C. Proteins were then eluted with 2 ml of the elution buffer (200 mM imidazole, 50 mM Tris-HCl, pH 8.0, and 100 mM NaCl) at 4°C and dialyzed against a storage buffer (50 mM Tris-HCl, pH 7.6, 200 mM KCl, 10 mM MgCl_2_, 0.1 mM EDTA, 1 mM dithiothreitol, and 50% glycerol).

### Extraction and quantification of intracellular c-di-GMP.

Intracellular c-di-GMP levels were determined using high-performance liquid chromatography tandem mass spectrometry (HPLC-MS/MS), as previously described (66), with the following modifications. Strain P1 cells, obtained from OD-adjusted overnight precultures, were inoculated onto various PAH substrates and grown for 48 h in triplicate. Bacterial pellets were resuspended in 0.2% ice-cold formaldehyde at a concentration of 300 mg wet weight/ml, incubated on ice for 10 min, and then centrifuged at 10,000 × *g* for 15 min. Each pellet was then resuspended in 300 ml of H_2_O. After extracting nucleotides using ethanol extraction (67), samples were dried under a vacuum, reconstituted in 50 ml of H_2_O via vigorous vortexing and sonication for 5 min, and finally centrifuged for 15 min at 10,000 × *g*. The supernatants were analyzed at 55°C using an Agilent 1200 HPLC system with a C_18_ ACE 3 AQ 150- by 2.1-mm column and the corresponding column guard. The mobile phase consisted of 0.1% formic acid run in an H_2_O-acetonitrile gradient at a flow rate of 300 μl/min, reaching 50% acetonitrile in 15 min. MS analysis was then performed via negative ion electrospray from the Agilent HPLC system into a Bruker HCT Plus ion trap in multiple reaction mode. Using SmartFrag (HyStar software), the trap was set to isolate fragment ions at *m/z* 688.9 (with a monitored mass range of *m/z* 450 to 700) from a full scan. Data Analysis v3.3 was used to interrogate the acquisitions, and the extracted ion chromatograms of *m/z* 538 were produced from the negative-ion MS/MS of *m/z* 688.9. The retention times and peak spectra were matched to the injected standard (c-di-GMP; Biolog LifeScience Institute, Bremen, Germany) at intervals throughout the run. Peak areas and relative c-di-GMP levels were normalized to the levels obtained with the injected standard. A solution of 1 mg/ml c-di-GMP standard in water was diluted to prepare the spiking solutions with 100 ng/ml, 200 ng/ml, 300 ng/ml, 400 ng/ml, 500 ng/ml, 600 ng/ml, 700 ng/ml, 800 ng/ml, and 900 ng/ml c-di-GMP standard. Extractions and quantifications were all performed in triplicate. The amount of c-di-GMP detected in the blank was subtracted from that detected in the spiked calibrants, and the analysis was calibrated using a linear regression (*r*^2^ = 0.993). To obtain an estimate of c-di-GMP concentration per unit of cellular biomass, the c-di-GMP concentration was divided by the measured OD_600_ of the original culture.

### PdgC enzymatic activity assay.

Assays were performed using the Enzchek pyrophosphate assay kit (Invitrogen). The concentration of MgCl_2_ was increased to 2 mM (final concentration), and GTP (USB) was used at 0.5 mM (final concentration). In addition, the pyrophosphatase in the kit was replaced by 0.4 U/ml pyrophosphatase (Sigma). To test for PdgC activity, PdgC (at concentrations ranging from 30 nM to 3.0 μM) was equilibrated in reaction buffer overnight at 22°C, and PAHs (naphthalene, phenanthrene, and pyrene) were dissolved in DMSO to a final concentration of 2 μM. The kit enzymes and pyrophosphatase were added to the reaction mixtures before the reaction with GTP was initiated. The accumulation of phosphate was monitored over time at OD_360_.

### SPR experiments in PdgC.

The immobilization of the PdgC PAS domain on the sensor surface was carried out as previously described (68). Briefly, PdgC was diluted in 10 mM sodium acetate (pH 5.5) and immobilized on a CM5 sensor chip using the amine coupling method by following the manufacturer’s protocol. SPR measurements were performed using a Biacore T200 system (GE Healthcare, Sweden).

PAH compounds were diluted in running buffer (phosphate-buffered saline [PBS] containing 5% DMSO) to a concentration of 50 μM. Running buffer alone (PBS containing 5% DMSO, without PAHs) served as the negative control. All solutions were then injected into the PdgC sensor surface for 120 s at a flow rate of 30 μl/min. Sensograms of these PAHs were recorded and analyzed. Analytes were injected through the reference and active channels at a flow rate of 30 μl/min. The association and dissociation times were both 120 s. Affinity fitting (determination of the affinity constant, *K_D_*) was performed using Biacore T200 evaluation software with a 1:1 binding mode.

### SPR experiments in DPRs.

We covalently immobilized 50 μg/ml DPR-1 and DPR-2 in 10 mM sodium acetate buffer (pH 5.5) onto separate CM5 sensor chips (GE Healthcare), using standard primary amine coupling procedures. SPR experiments were performed using a Biacore T200 instrument (GE Healthcare) at 25°C, with a flow rate of 30 μl/min. After testing different buffer conditions for DPRs, the assay buffer selected was 20 mM morpholinepropanesulfonic acid (MOPS), pH 8.0, 300 mM NaCl, and 0.005% P20 with 0.1 mg/ml bovine serum albumin (BSA). Direct binding assays with immobilized DPR-1 or DPR-2 were performed by injecting increasing concentrations (5, 10, and 20 mM) of c-di-GMP or cAMP (negative control) into each sensor chip. After double referencing by subtracting the signal of the reference flow cell and the curve of the buffer injection, *K_D_* values for c-di-GMP binding to the DPRs immobilized on the sensor chip surface were calculated using reference points at the end of the association phase. Values were calculated with Biacore T200 evaluation software using a 1:1 binding mode.

The experiments were carried out in a running buffer composed of 20 mM Tris-HCl, 150 mM NaCl, and 0.005% Tween 20 (pH 7.0), with a flow rate of 30 μl/min. We immobilized 5′-biotinylated double-stranded DNA fragments of various promoters on separate SA sensor chips (GE Healthcare) with the promoter of the permease gene *perT*2 (gene Q91_0869) and the promoters of various operons (genes Q91_0870 to Q91_0877, pyrene-degrading; genes Q91_2218 to Q91_2220, genes Q91_2224 to Q91_2227, and genes Q91_2242 to Q91_2244/PQ91_2242 to Q91_2244). A DNA fragment of the Q91_1174 promoter was used as a negative control. His6-tagged DPR-1 or DPR-2, with or without c-di-GMP ligands, was diluted in running buffer to construct solutions of different concentrations. These solutions were individually injected using the K-inject command. At the end of each cycle, 0.05% SDS was used to regenerate the surface of the sensor chip. The data were fit to the 1:1 binding model using Biacore T200 evaluation 2.0 software (GE Healthcare).

### Construction of 3×FLAG epitope-tagged proteins in *Cycloclasticus* sp. strain P1.

*Cycloclasticus* sp. strain P1 genes were chromosomally tagged at their C termini by cloning of constructs for C-terminal 3×FLAG epitope tagging on plasmids. The DPR-1 and DPR-2 proteins were fused to a 3×FLAG epitope at each C terminus by cloning regions encoding about 500 bp of the C-terminal coding region (C-terminal but without the stop codon) and about 500 bp downstream of the stop codon into plasmid pET-3×Flag to flank a 3×FLAG tag and a Kan^r^ cassette (see Fig. S13 at https://doi.org/10.6084/m9.figshare.15042720.v1). Afterwards, the 3×FLAG tag constructs were amplified using PCR and introduced into the chromosomes of *Cycloclasticus* sp. strain P1 by electroporation and double-crossover homologous recombination. Approximately 500 bp of the C-terminal coding region of DPR-1 (without the stop codon) and ∼500 bp of the region downstream of the DPR-1 stop codon were amplified from the gDNA using the primers DPR1-1f/DPR1-1r and DPR1-2f/DPR1-2r, respectively. The primers DPR1-1f/DPR1-1r and DPR1-2f/DPR1-2r included the BamHI/HidIII and XhoI/PstI sites, respectively. The amplified upstream PCR product (i.e., the region upstream of the DPR-1 C-terminal coding region without the stop codon) was digested with BamHI and HindIII and then ligated into a similarly digested pET-3×Flag backbone to create pET-3×Flag-1. Next, the amplified downstream PCR product (i.e., downstream of the DPR-1 stop codon) was digested with XhoI and PstI and then ligated into a similarly digested pET-3×Flag-1 backbone to create pET-3×Flag-2. Finally, the entire integration cassette, including the C-terminal coding region of PahS (about 500 bp) without the stop codon, the 3×FLAG tag, and the region downstream of the DPR-1 stop codon, was amplified using PCR with primers petf/petr from the backbone of pET-3×Flag-2 plasmid. The amplified product was electroporated into *Cycloclasticus* sp. strain P1. Mutants were confirmed by Western blot analysis with an anti-FLAG antibody. A similar plasmid cloning strategy, which included DPR-1, DPR-2, and related primer sequences, is described in Table S4 at https://doi.org/10.6084/m9.figshare.15042720.v1.

For electroporation, strains grown from frozen stocks until passage one or two on modified ML solid medium were harvested into cold electroporation buffer (272 mM sucrose, 15%, vol/vol, glycerol) and washed twice with the same buffer. Cells (50 μl) were mixed with 200 to 400 ng PCR product on ice and electroporated using a Bio-Rad MicroPulser in a 1-mm gap cuvette (PEQLAB) at 2.5 kV. Cells were then transferred with Brucella broth to a modified ML solid plate and aerobically recovered overnight at 37°C before plating on the appropriate selective medium.

### ChIP-seq.

The ChIP protocol was based on previously published methods (69) and the Affymetrix ChIP assay protocol, with several modifications. The two mutated strains (*dpr*-1-3×Flag and *dpr-*2-3×Flag) and wild-type strain P1 were separately cultured in 200 ml of ASM medium, supplemented with 500 ppm PAHs or sodium acetate, for 5 days (OD_600_ of 1.5). After induction with urocanic acid, formaldehyde was added to a final concentration of 1% (vol/vol), and the mixtures were incubated for 15 min at room temperature with gentle agitation. Crosslinking was halted by the addition of glycine to a final concentration of 125 mM, and bacteria were subsequently washed three times in ice-cold PBS buffer. Bacterial pellets were resuspended in the lysis buffer (50 mM HEPES, pH 7.5, 150 mM NaCl, 1 mM EDTA, 1% [vol/vol] Triton X-100, 0.1% [wt/vol] sodium deoxycholate [DOC], 0.1% [wt/vol] SDS, complete protease inhibitor cocktail [Roche], 1 mM phenylmethylsulfonyl fluoride [PMSF]), and chromatin was fragmented using a sonicator (Misonix XL-2000) in an ice bath, with 12 pulses of 30 s each at 1.5-min intervals. DNA was sheared by sonication to an average size of 100 to 1,000 bp. The supernatant was clarified at 13,000 rpm for 10 min at 4°C. Each immunoprecipitation (IP) reaction mixture contained a 200-μl aliquot of the input sample, 800 μl of IP buffer (50 mM HEPES-KOH, pH 7.5, 150 mM NaCl, 1 mM EDTA, 1% Triton X-100, and 1 mM PMSF), and 40 μl EZ-view anti-FLAG agarose beads (Sigma) (equilibrated in Tris-buffered saline, TBS). IP was performed for 2 h at room temperature on a rotator. Following immunoprecipitation, beads were collected and washed by following the Affymetrix ChIP assay protocol. Immunoprecipitated complexes were eluted, and cross-links were reversed as described in the Affymetrix ChIP assay protocol. Samples (input DNA and IP DNA) were analyzed using qRT-PCR to assess the quality of the immunoprecipitates from three independent experiments. DNA from representative ChIP samples was prepared for sequencing using the Illumina ChIP-seq sample preparation kit. The following ChIP samples were sequenced: the input DNA and IP DNA from the *pahR*-3×Flag strain (PAHs or sodium acetate induction, respectively), the input DNA and IP DNA from the *dpr*-1-3×Flag strain (PAHs and sodium acetate induction, respectively), the input DNA and IP DNA from the *dpr*-2-3×Flag strain (PAHs and sodium acetate induction, respectively), and the input DNA and IP DNA from the wild-type strain P1 (PAHs or sodium acetate induction, respectively).

### ChIP-seq data analysis. (i) Aligning sequence reads.

Sequence reads were aligned to the reference genome of *Cycloclasticus* sp. strain P1 (GenBank accession number CP003230) using Bowtie version 0.12.8 (70).

### (ii) Peak calling.

To identify peaks representing putative DPR-1 and DPR-2 binding sites, all uniquely mappable reads were analyzed with CSDeconv (71). CSDeconv was used because it is the only currently available tool capable of distinguishing closely spaced binding sites (fewer than 100 bp apart) in ChIP-Seq data.

### (iii) Motif identification.

DPR-1 and DPR-2 binding motifs were identified using Multiple Em for Motif Elicitation (MEME) version 4.8.1 (72). The set of DNA sequences to be analyzed was derived by extracting 80 nucleotides upstream and downstream of each predicted peak position (161 bp in total) and merging overlapping regions.

### Real-time PCR.

*Cycloclasticus* sp. strain P1 and mutant strain PARP1 were grown in 200 ml of MMC medium containing the appropriate antibiotics, with sodium acetate or PAHs as the carbon source, in 500 ml Erlenmeyer flasks at 28°C on a rotary shaker (200 rpm). Bacterial growth was monitored based on optical density (OD_600_). Total RNA was extracted using the RNeasy minikit (Qiagen, Valencia, CA, USA) according to the manufacturer’s protocols and subsequently treated with DNase I (Invitrogen, Carlsbad, CA, USA). The RNA yield was estimated using a NanoDrop UV spectrometer (Thermo Scientific, Wilmington, DE, USA). Approximately 4 μg of RNA was reverse transcribed using 20 ng of random primers (Invitrogen, Carlsbad, CA, USA) and PrimeScript reverse transcriptase (TaKaRa, Dalian, China). Control reactions were performed in the absence of reverse transcriptase to verify the absence of genomic DNA. To calculate the number of viable bacteria present, 16S rRNA gene copy numbers were determined using quantitative real-time PCR. The 16S rRNA gene copy numbers ranged from approximately 10^7^ to approximately 10^9^ in each sample.

The gene-specific primer sequences (Table S4 at https://doi.org/10.6084/m9.figshare.15042720.v1) were synthesized by Invitrogen (Shanghai, China). Quantitative real-time PCRs were performed using IQ SYBR green Supermix and the IQ5 multicolor real-time PCR detection system (Bio-Rad, CA, USA). The reactions were performed in 96-well optical plates sealed with optical caps. Each 25-μl reaction volume contained 12.5 μl of 2× SYBR green PCR supermix (Bio-Rad, CA, USA), the DNA template, primers at optimized concentrations, and sterile water. The PCR cycling conditions were 2 min at 50°C (uracil-N-glycosylase activation), 10 min at 95°C (activation of *Taq* polymerase), and 45 cycles of denaturation (10 s at 95°C), annealing, and elongation (30 s at 56 to 61°C). Fluorescence data were acquired at the end of the elongation step. The specificities of the accumulated products were verified using melting curve analysis. In all experiments, the appropriate negative controls were subjected to the same procedures to detect possible contamination. The size and purity of each amplicon, as well as the absence of dimer formation, were assayed using conventional agarose gel electrophoresis.

The samples were quantified using a calibration curve that was generated in parallel. A standard quantification curve was generated for each of the tested genes based on a serial dilution of a known amount of target DNA (from 10 to 10^6^ copies) utilizing the optimal PCR conditions described above. Each dilution was assayed in triplicate to generate each data point. For each standard, the concentration was plotted as a function of the cycle number at which the fluorescent signal increased above the background cycle threshold (*C_T_* value of 0.1). The slope of each calibration curve was used in the following equation to determine reaction efficiency: efficiency = 10^−1/slope^ − 1. An efficiency of 1 signified that the concentration of the product doubled every cycle. Based on the calibration curve (determined using IQ^5^ multicolor real-time PCR detection system software), the initial copy number of each target mRNA was calculated for each sample. The calculated values were normalized to the expression of the 16S rRNA gene to yield the number of copies of each mRNA transcript in 1,000 wild-type *Cycloclasticus* sp. strain P1 or mutant cells.
