# Supplementary figures and images for: An Intracellular Sensing and Signal Transduction System That Regulates the Metabolism of Polycyclic Aromatic Hydrocarbons in Bacteria
Source: mSystems. 2021 Oct 5;6(5):e00636-21. doi: 10.1128/mSystems.00636-21 (PMC8547461; doi:10.1128/mSystems.00636-21)

## Growth curve with pyrene

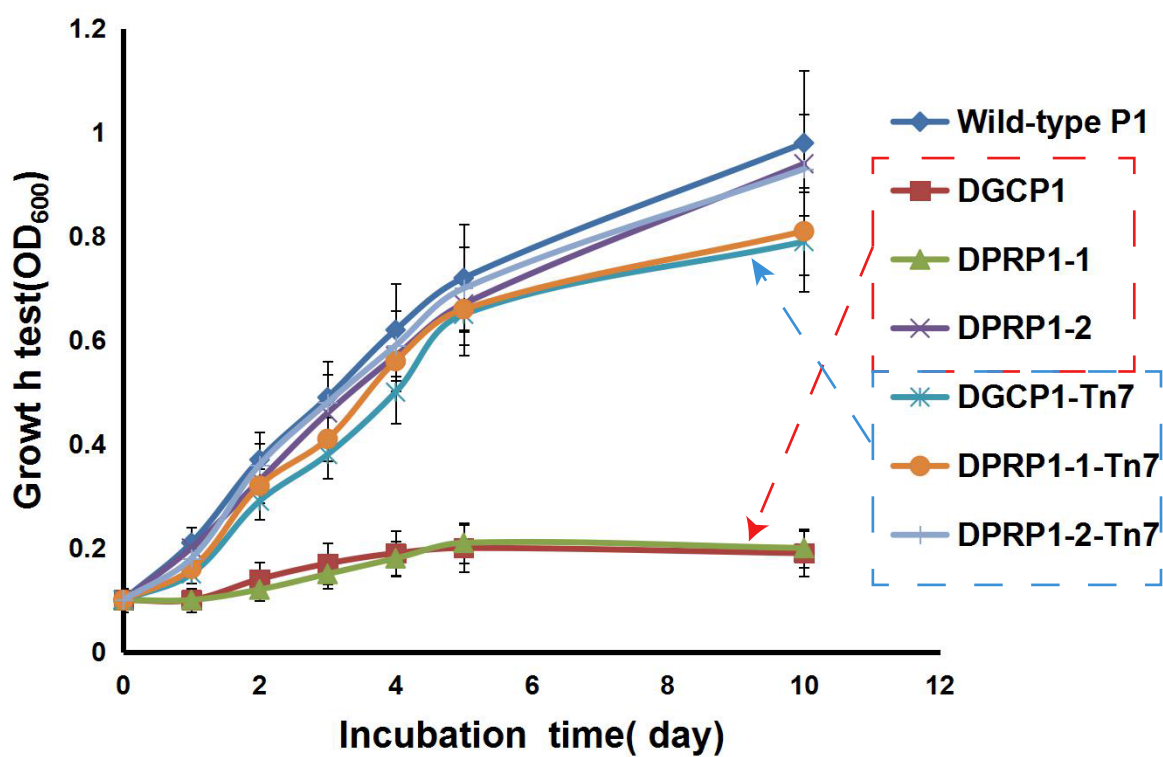

## Growth curve with phenanthrene

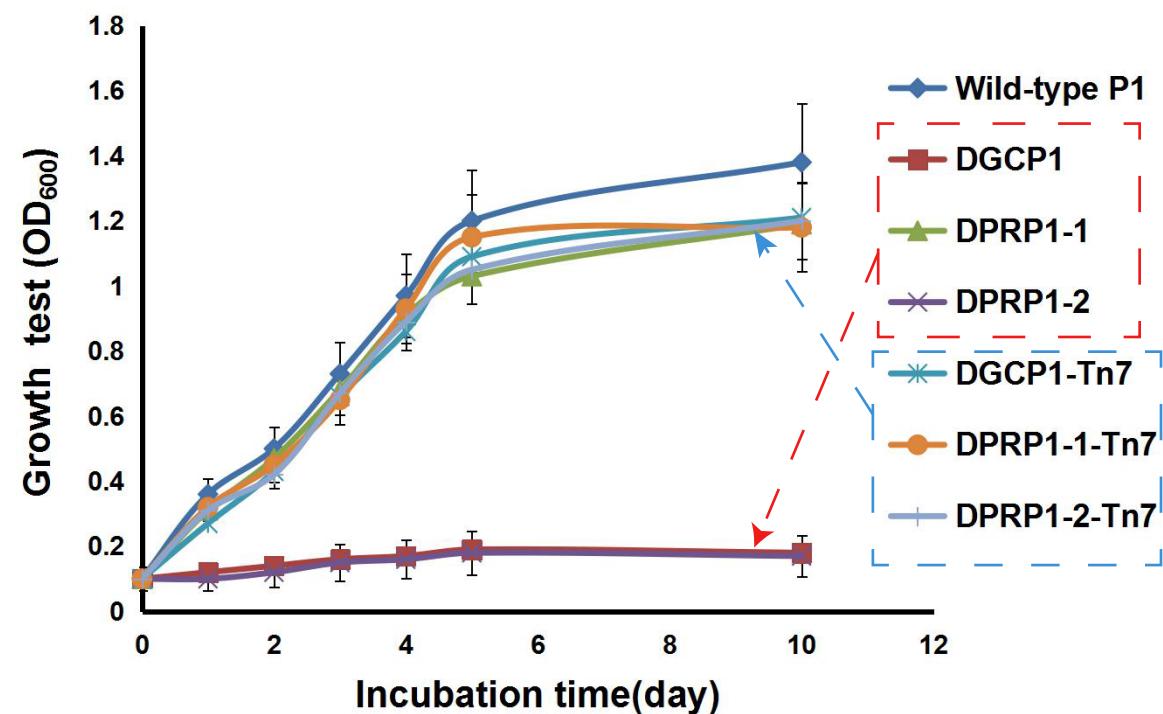

## Growth curve with naphthalene

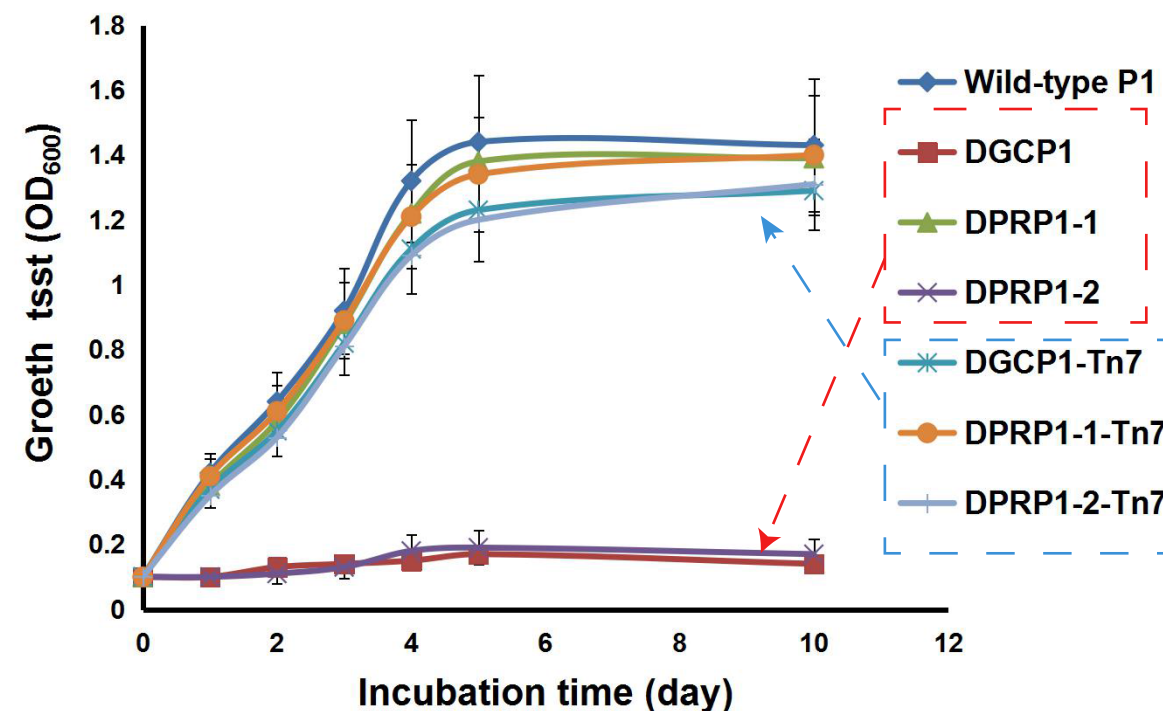

Supplement: FIG S1 [file msystems.00636-21-sf001.pdf]

# A

## DGC

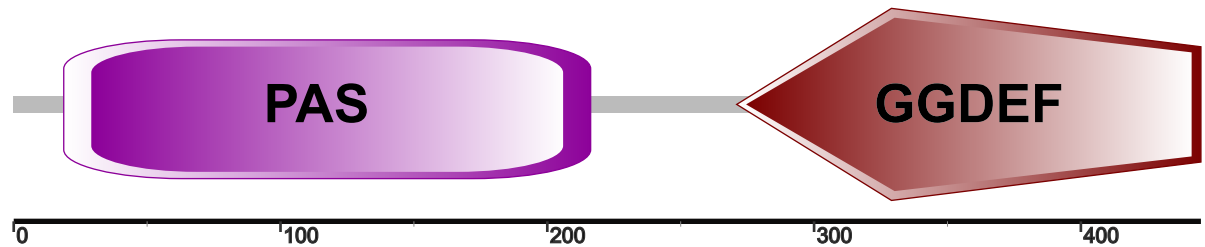

# B

## DPR-1

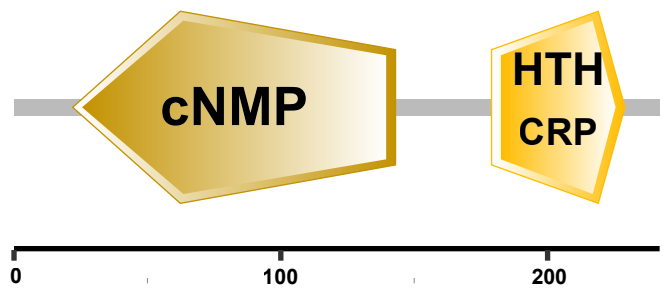

## DPR-2

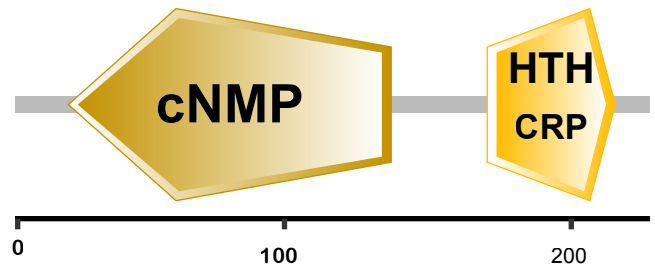

Supplement: FIG S2 [file msystems.00636-21-sf002.pdf]

### Naphthalene

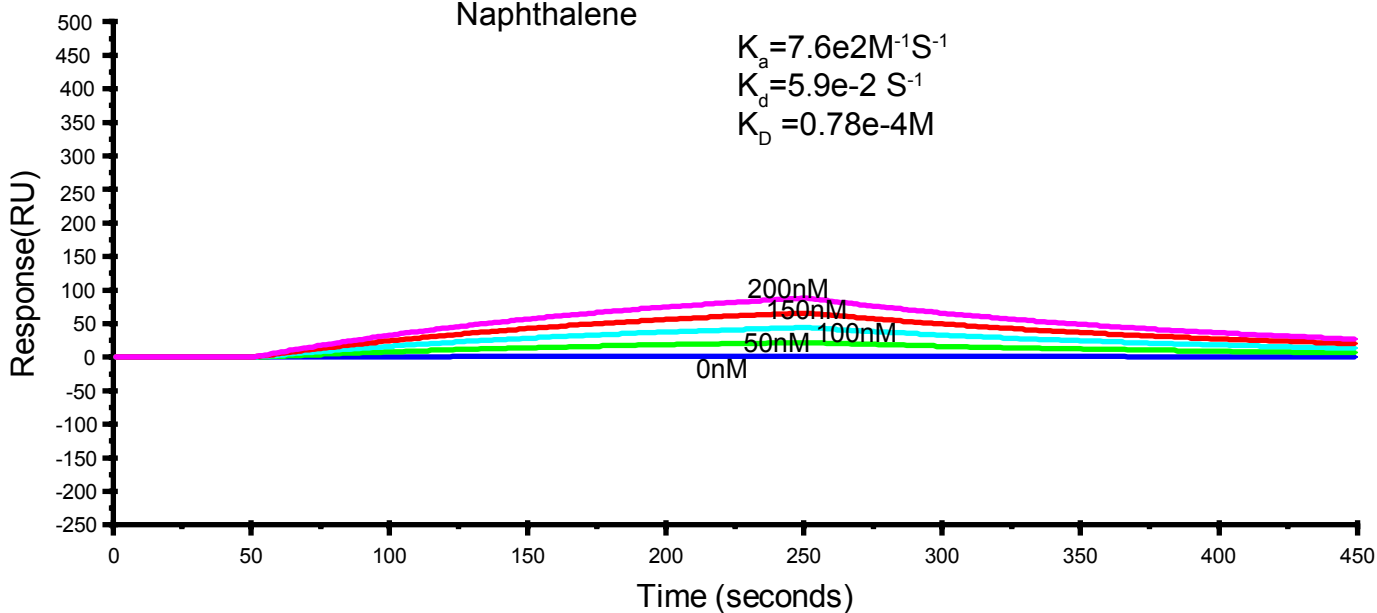

### Phenanthrene

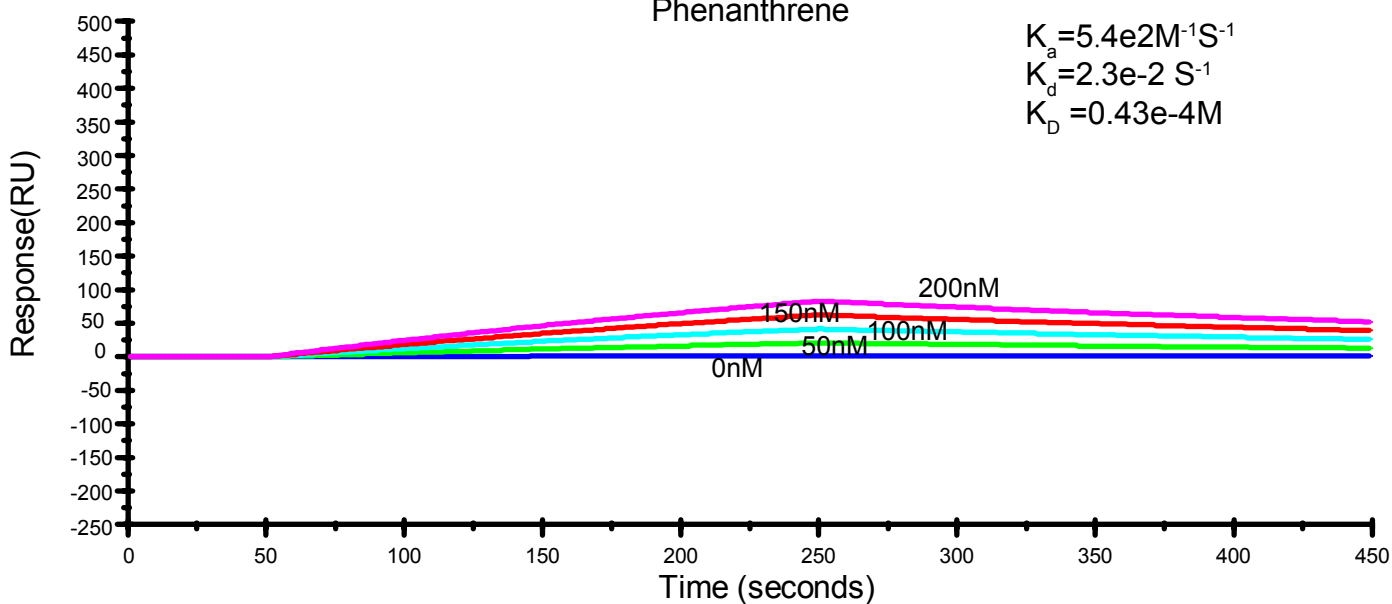

### Pyrene

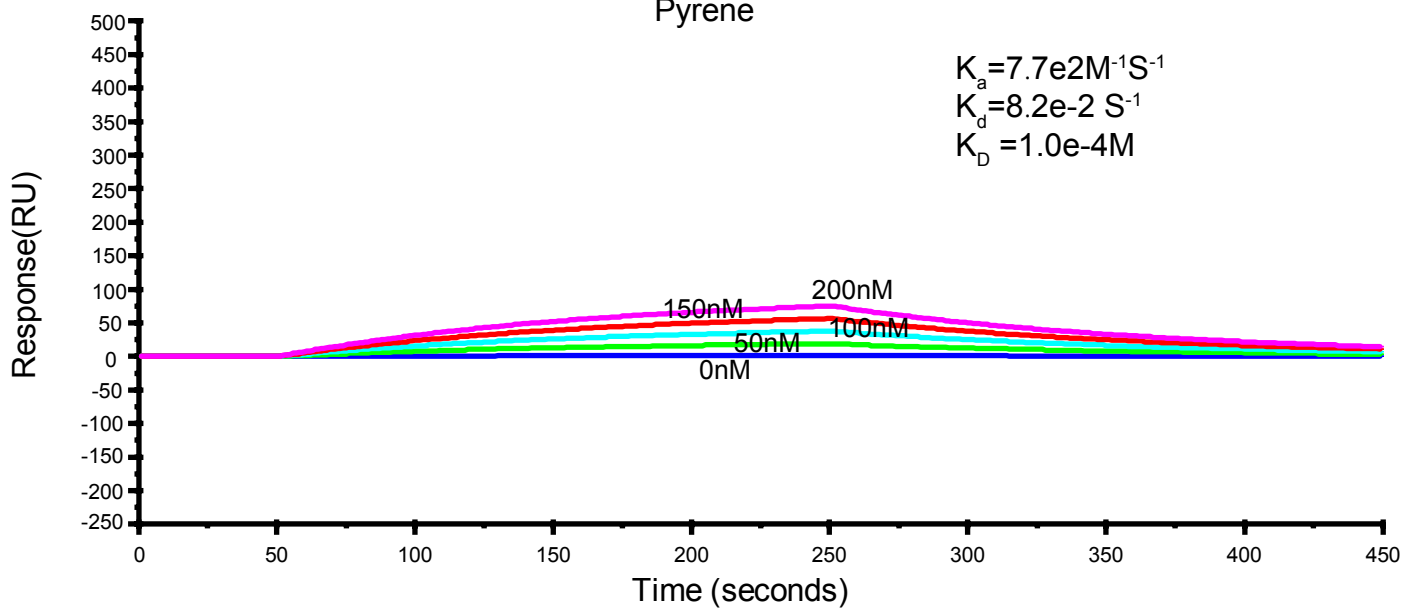

Supplement: FIG S3 [file msystems.00636-21-sf003.pdf]

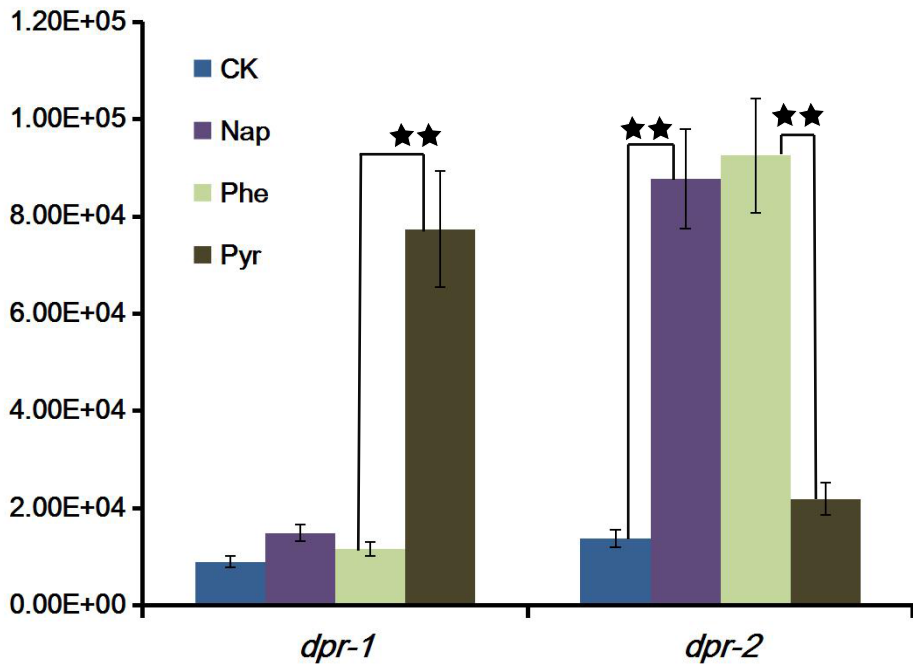

Supplement: FIG S4 [file msystems.00636-21-sf004.pdf]

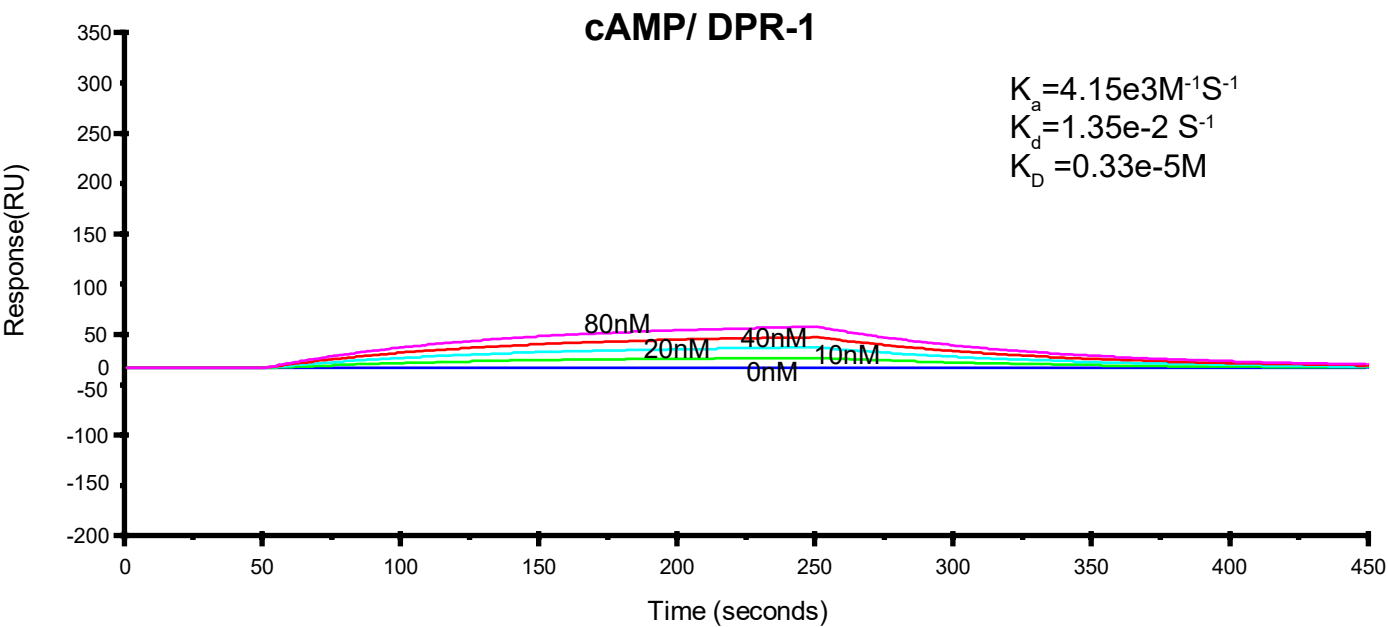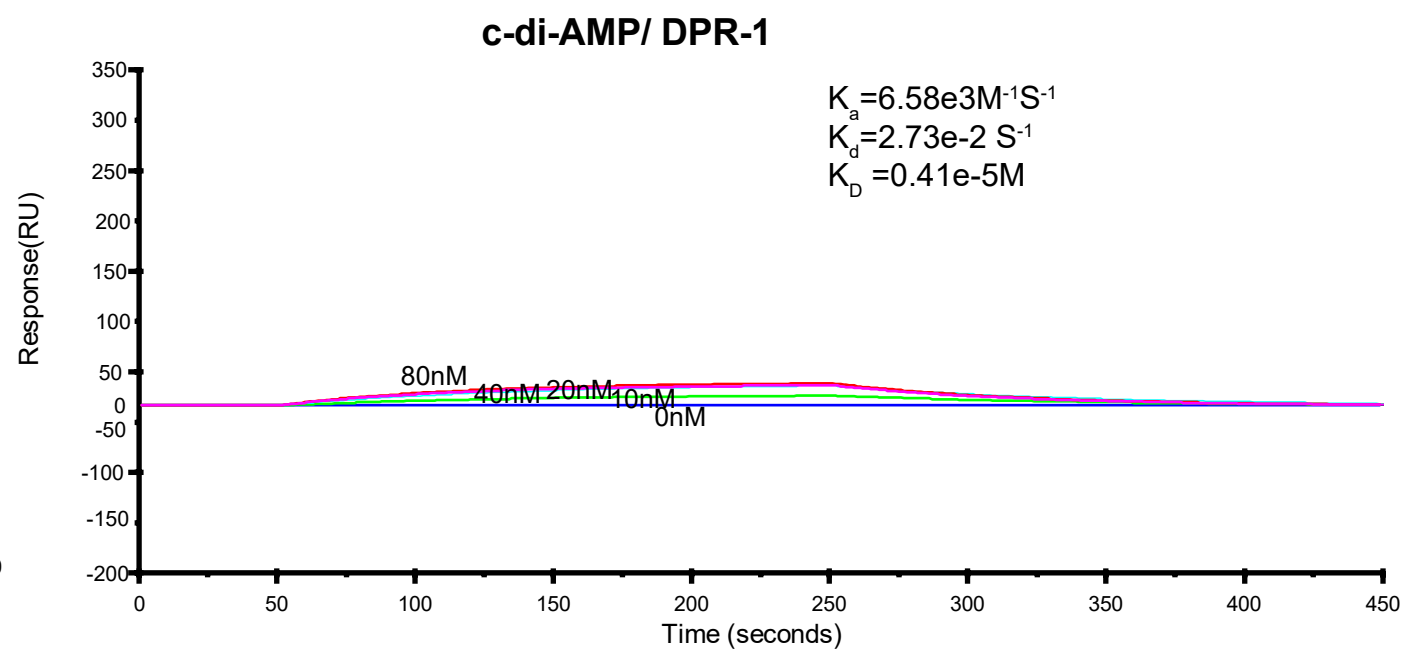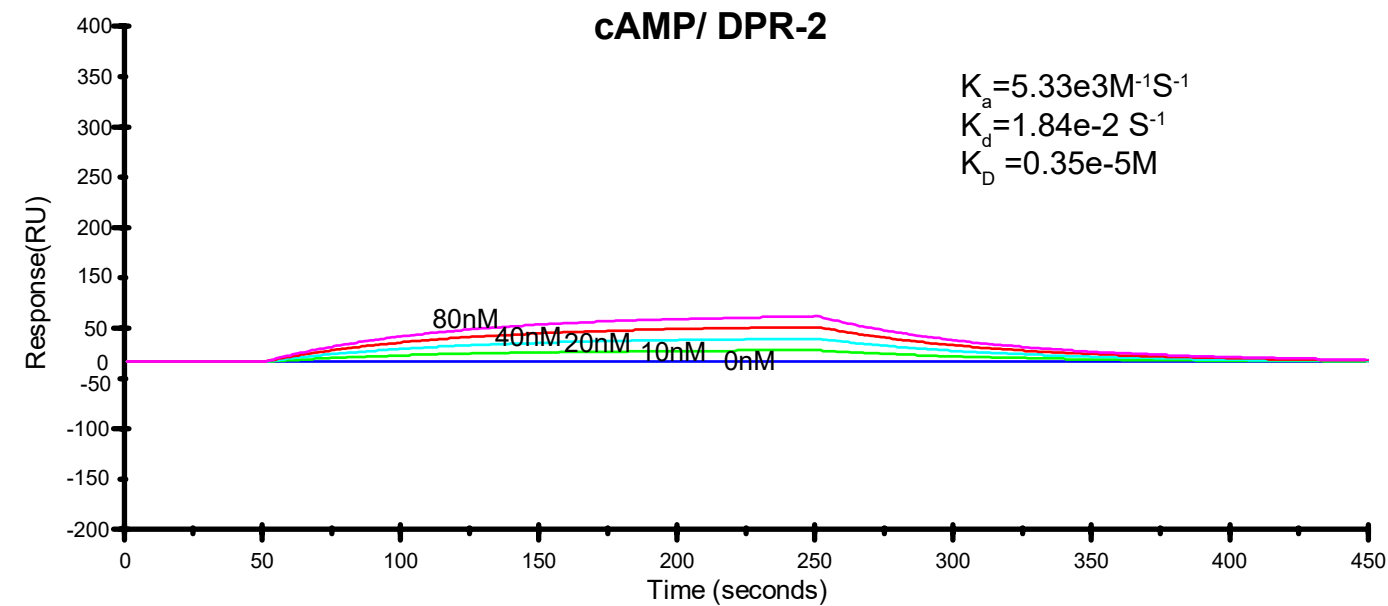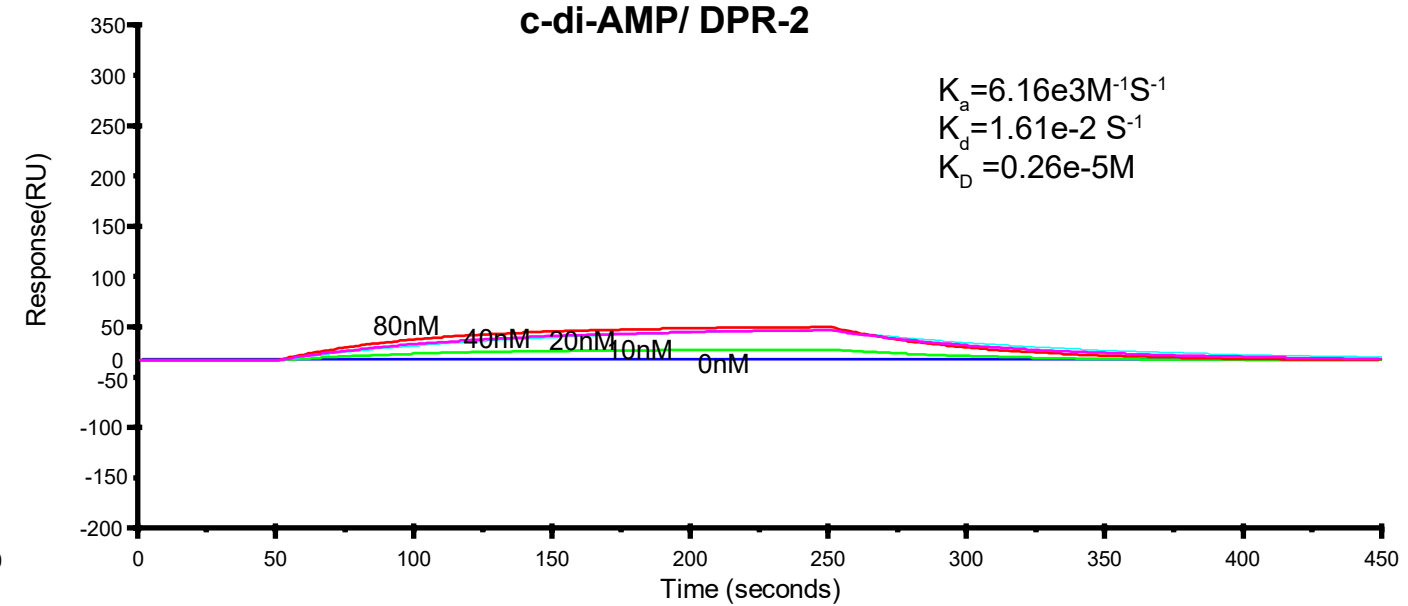

Supplement: FIG S7 [file msystems.00636-21-sf007.pdf]

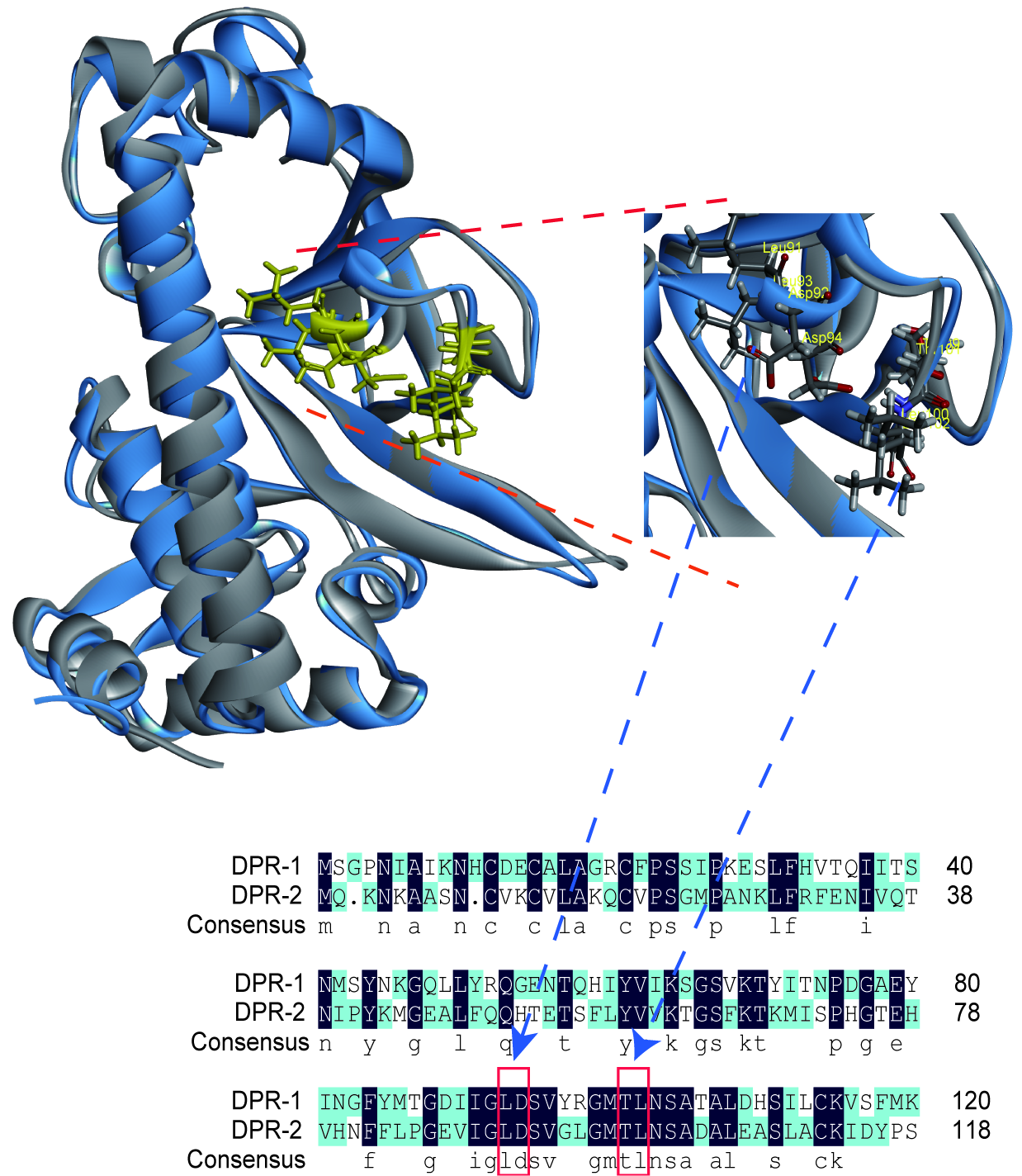

Supplement: FIG S8 [file msystems.00636-21-sf008.tif]

**A****Wild-type P1 strain**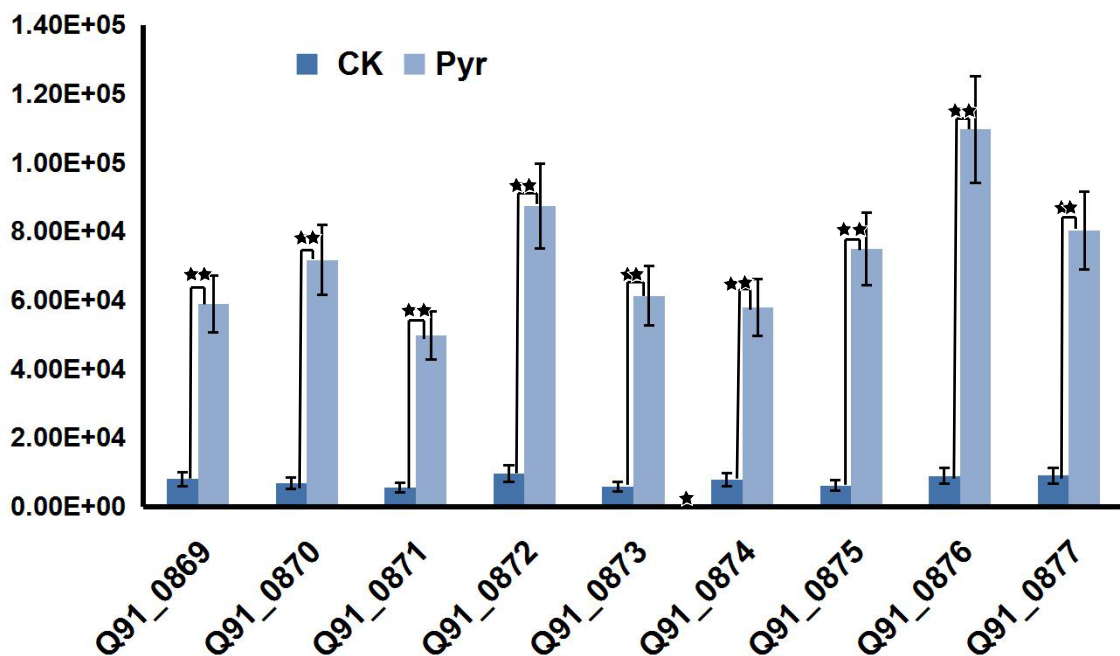**B****DPRP1-1 mutant strain**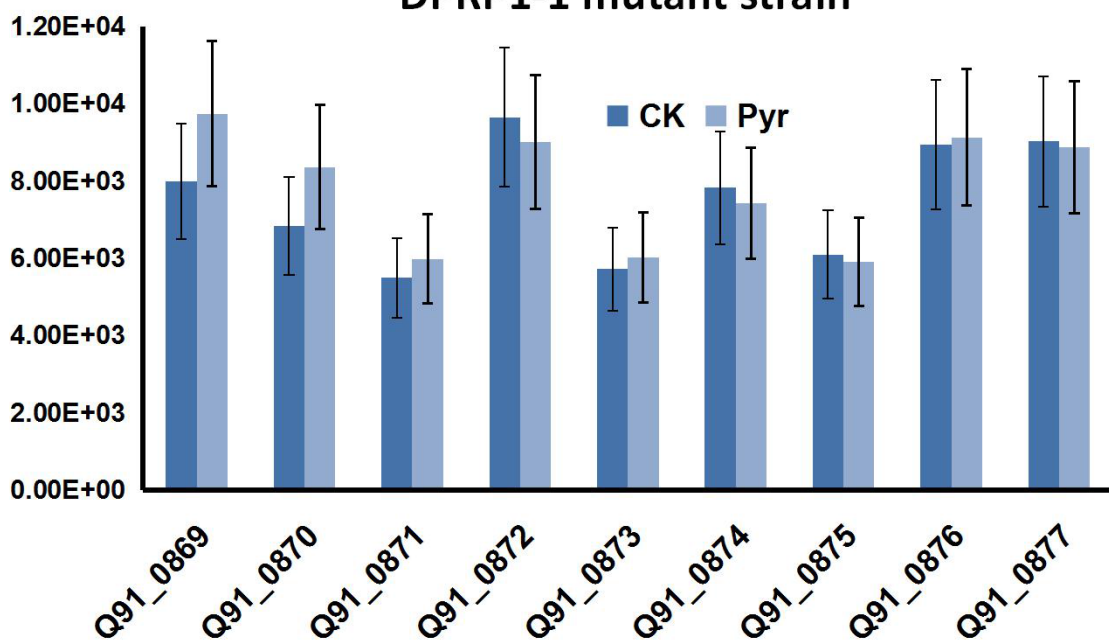**C****PdgC mutant strain**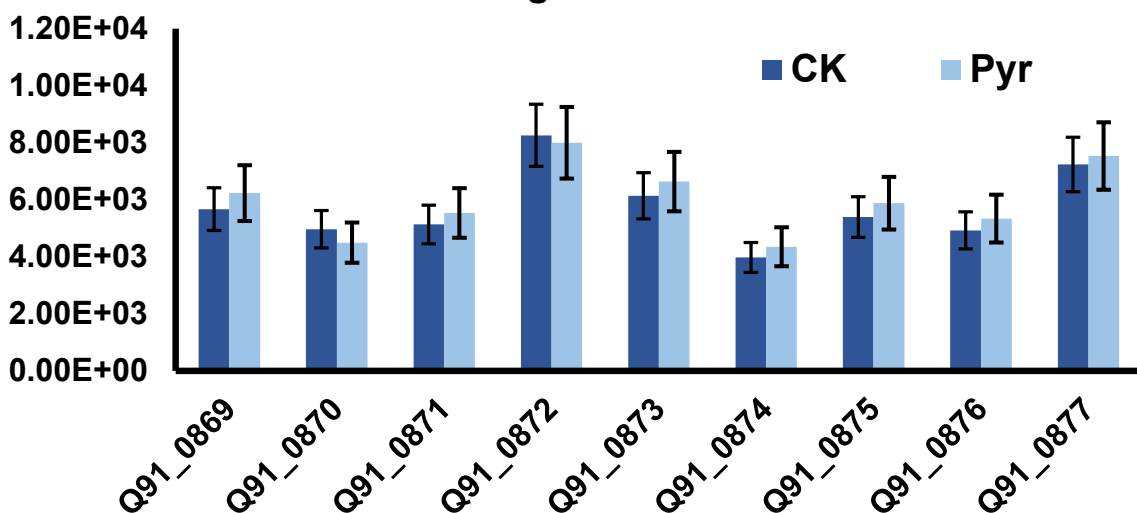

Supplement: FIG S5 [file msystems.00636-21-sf005.pdf]

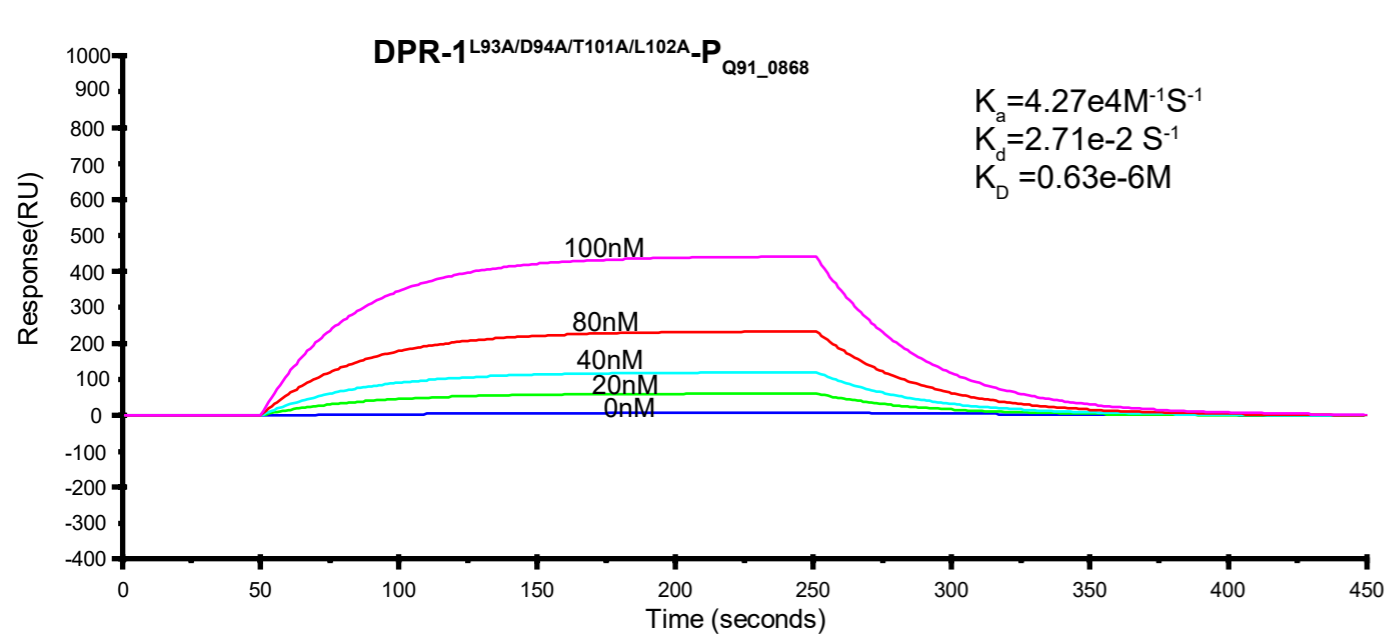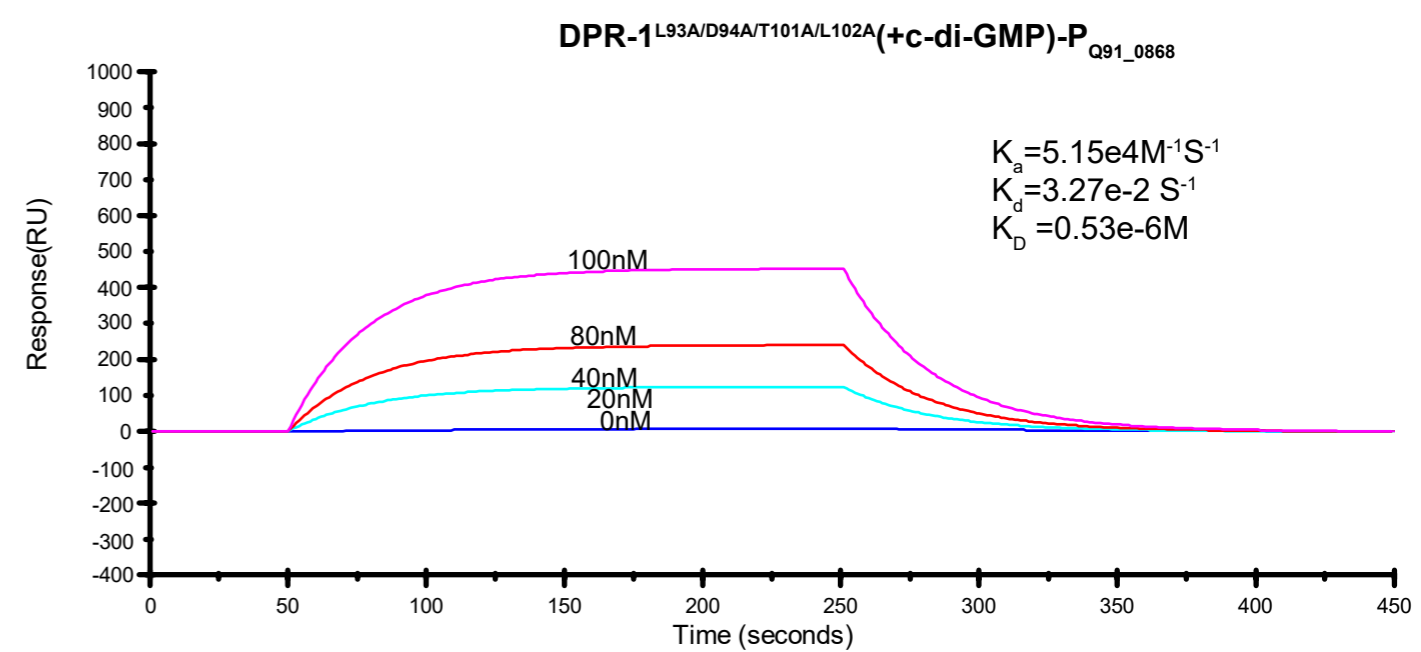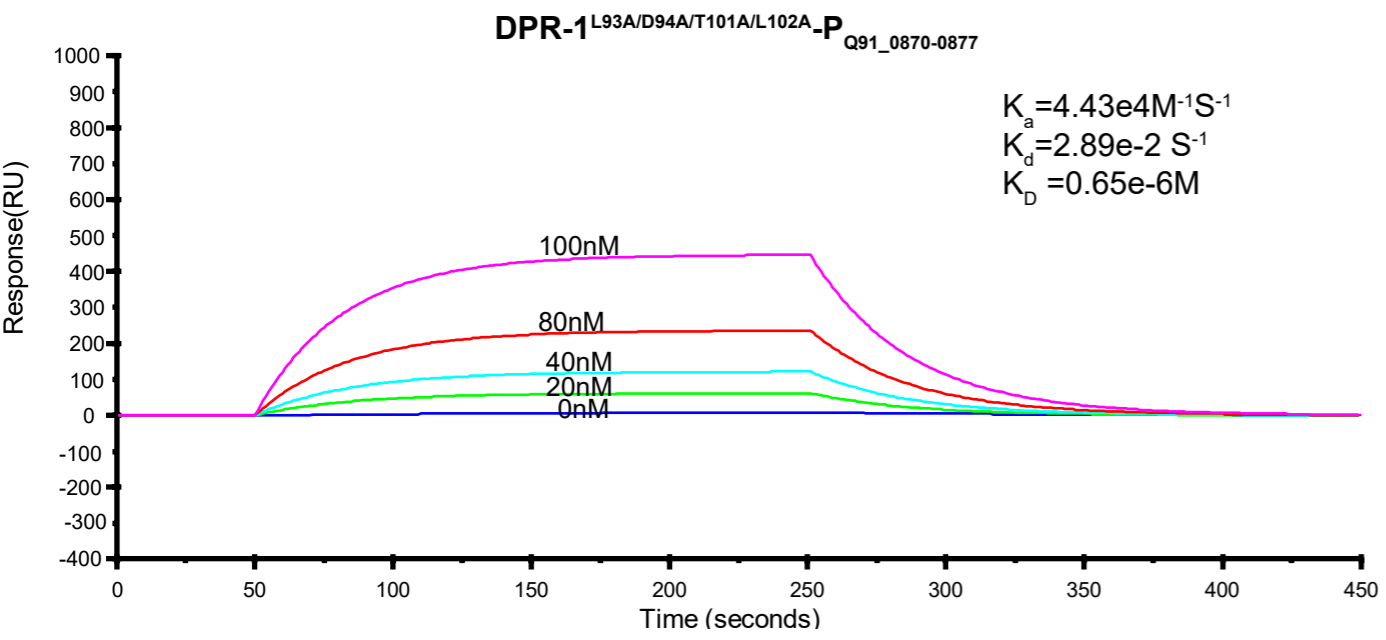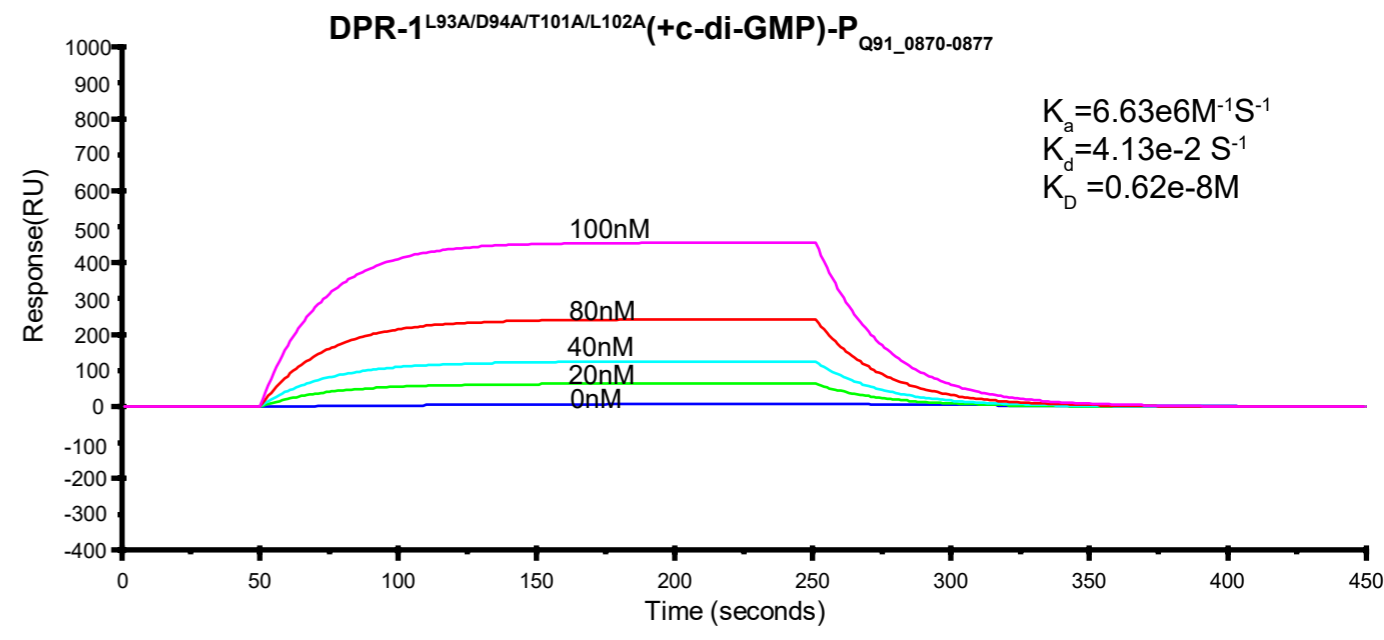

Supplement: FIG S9 [file msystems.00636-21-sf009.pdf]
