# Supplementary material for: An Intracellular Sensing and Signal Transduction System That Regulates the Metabolism of Polycyclic Aromatic Hydrocarbons in Bacteria
Source: mSystems. 2021 Oct 5;6(5):e00636-21. doi: 10.1128/mSystems.00636-21 (PMC8547461; doi:10.1128/mSystems.00636-21)
Supplement: FIG S6 [file msystems.00636-21-sf006.pdf]

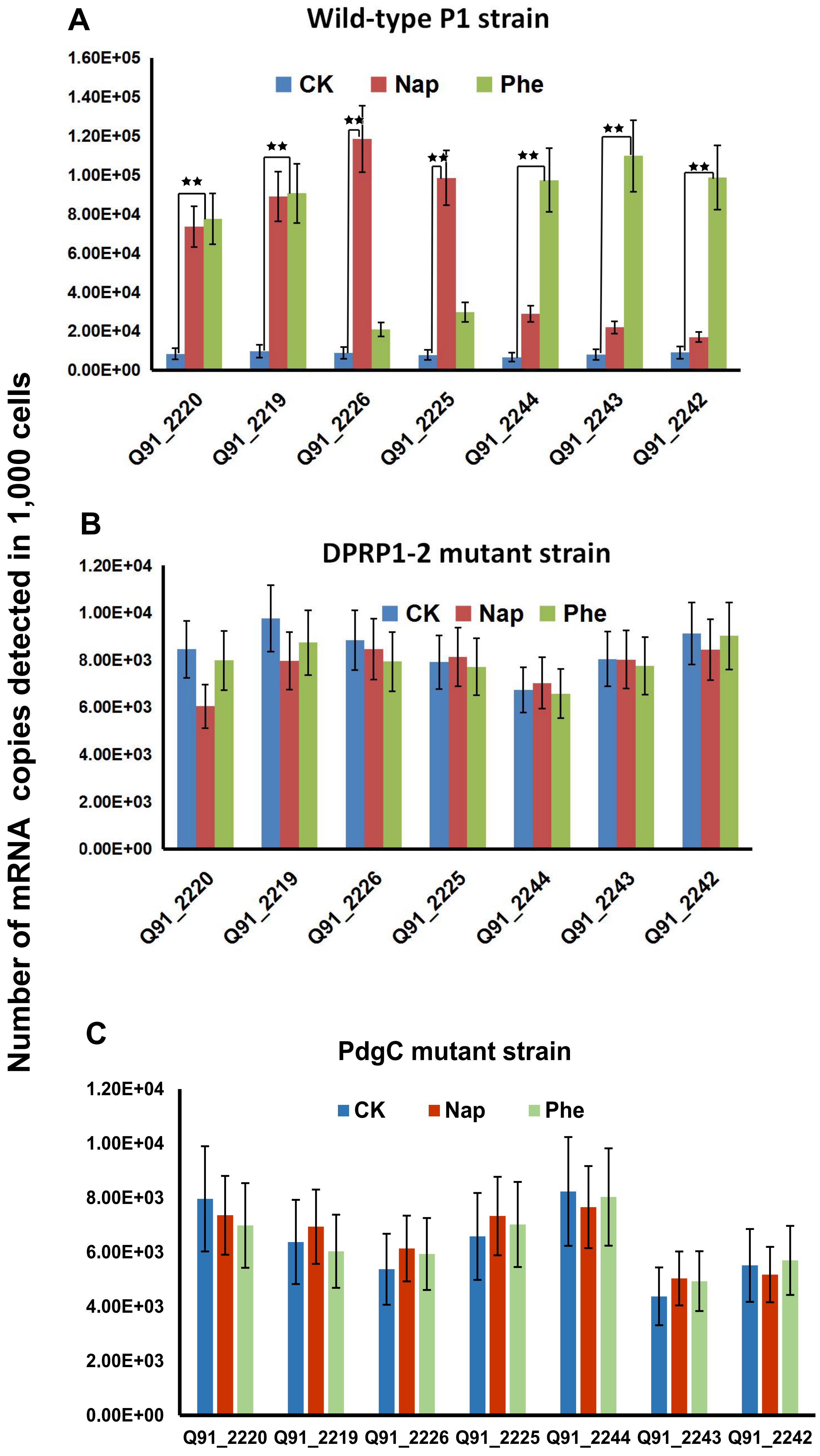

A
Wild-type P1 strain
B
DPRP1-2 mutant strain
C
PdgC mutant strain
CK
Nap
Phe
Number of mRNA copies detected in 1,000 cells
1.60E+05
1.40E+05
1.20E+05
1.00E+05
8.00E+04
6.00E+04
4.00E+04
2.00E+04
0.00E+00
1.20E+04
1.00E+04
8.00E+03
6.00E+03
4.00E+03
2.00E+03
0.00E+00
Q91_2220
Q91_2219
Q91_2226
Q91_2225
Q91_2244
Q91_2243
Q91_2242
